# Smart crop recommendation: fusing nutrient and climate data with Krill Herd Optimization and explainable AI

**DOI:** 10.3389/frai.2026.1910196

**Published:** 2026-09-09

**Authors:** P. Latha, P. Kumaresan

**Affiliations:** 1 School of Computer Science Engineering and Information Systems (SCORE), Vellore Institute of Technology, Vellore, India; 2 Centre For Artificial Intelligence Research (C-FAIR) Vellore Institute of Technology, Vellore, India

**Keywords:** crop recommendation system, explainable artificial intelligence (XAI), Krill Herd Optimization (KHO), LIME, multi-layer perceptron (MLP), nutrient-based crop prediction, precision agriculture, SHAP

## Abstract

Crop recommendation is a vital part of precision agriculture as it helps farmers choose appropriate crops according to the nutrient profile and environmental conditions. This paper presents a crop recommendation framework in which Multi-Layer Perceptron (MLP), XGBoost, and Tab Transformer are first evaluated as baseline prediction models, followed by the proposed Krill Herd Optimization (KHO)-based explainable framework integrated with Explainable Artificial Intelligence (XAI). These eleven parameters are created based on agronomic and environmental aspects, namely: Nitrogen, Phosphorus, Potassium, Copper, Iron, Magnesium, Sulphur, Temperature, Rainfall, pH and Humidity. For better model transparency and to aid informed decision-making, model explanations with SHAP (SHapley Additive Explanations) and LIME (Local Interpretable Model-Agnostic Explanations) are used to identify feature contributions to crop predictions both locally and globally. The experimental results showed that Tab Transformer significantly outperformed the other models, with an accuracy of 0.99, precision of 0.98, recall of 0.99 and F1-score of 0.98. The proposed framework further incorporates Krill Herd Optimization (KHO) to generate optimized nutrient and climate profiles, while Genetic Algorithm (GA), Particle Swarm Optimization (PSO), and Simulated Annealing (SA) are used for comparative evaluation of optimization performance. By combining explainable AI with optimization methods, the framework improves crop suitability prediction and provides transparent insights into the factors influencing crop recommendations, ensuring reliable decision support for practical farming applications. The proposed framework supports precision agriculture by enabling data-driven crop selection, reducing unnecessary fertilizer usage, optimizing crop productivity, and promoting sustainable farming practices.

## 1 Introduction

Crop selection in agriculture is challenging due to variability in soil nutrients, climate, and environmental conditions. Traditional methods rely on manual labor and historical knowledge, often leading to suboptimal productivity. While ML and DL-based approaches improve prediction accuracy, they lack interpretability, optimization ability, and fail to capture complex feature interactions. Therefore, an intelligent framework is needed that accurately models these interactions, enhances prediction accuracy, and supports transparent decision-making for precision agriculture.

One of the key components in precision agriculture is soil nutrient prediction and crop recommendations, which allow farmers to choose appropriate crops for their soil fertility and environmental conditions. The capacity to capture complex relationships between nutrient and climatic factors has advanced with recent advances in Artificial Intelligence (AI), specifically transformer-based learning. Additionally, the use of bio-inspired optimization methods has grown, as a means of optimizing model parameters and improving prediction. But most of the current methods focus mainly on the accuracy of prediction with little interpretability of the model. Thus, there is a proliferation of research need to develop intelligent frameworks that combine transformer-based learning, optimization, and Explainable Artificial Intelligence (XAI) to provide accurate, transparent and reliable crop recommendations.

The novelty is the combination of transformer-based learning, Krill Herd Optimization (KHO), and Explainable Artificial Intelligence (XAI) in a single framework that will improve the prediction accuracy, optimization, and model interpretability in precision agriculture. Current crop recommendation systems mostly concentrate on enhancing predictions accuracy by developing conventional machine learning and deep learning models, but many models lack interpretability and do not capture complex relationships among the agricultural features. The proposed framework is based on Tab Transformer to leverage a self-attention mechanism to model contextual relationships between nutrient and environmental variables for more effective learning of complex feature interactions, which is different from the previous approaches. To facilitate personalized crop recommendations based on real-time environmental and agronomic data adopting machine learning, this study introduces the Transformative Crop Recommendation Model (TCRM) based on cloud computing. This framework outperforms the traditional ones: its accuracy is 94%, the precision is 94.46%, the recall is 94% and the five-fold cross validation is 97.67%, making it effective for sustainable precision agriculture (Singh and Sharma, 2025). An AI-driven, IoT-integrated framework is proposed for unified crop and fertilizer recommendation, leveraging Tab Net to enable accurate, interpretable classification without manual feature selection. SHAP-based explainability, iterative imputation, and SMOTE are incorporated to improve transparency and data quality. The framework achieved 96.21% accuracy for crop recommendation and 95.24% accuracy for fertilizer recommendation, with performance validated through 5-fold cross-validation (Venkateswara and Padmanaban, 2025).

Furthermore, Optimization using Krill Herd (KHO) is built in to optimize model parameters and enhance the convergence behavior of the model, thus improving the performance of prediction and decreasing the prediction errors. In agriculture. Krill Herd Optimization (KHO), a bio-inspired swarm intelligence technique, has been implemented to optimize models and successfully used in agricultural applications such as crop monitoring, irrigation, and resource management, which improves the efficiency of decision-making (Maraveas et al., 2023). In addition, explainable AI methods like SHAP and LIME are integrated to increase transparency and uncover the set of parameters that drive crop recommendations.

Recent studies have applied deep learning for maize leaf disease detection; however, challenges such as similar disease symptoms, limited annotated datasets, and varying environmental conditions affect detection accuracy. To overcome these limitations, a customized CenterNet model based on the DenseNet-65 architecture was proposed, achieving simultaneous disease classification and localization with a classification accuracy of 98.62% on the CD&S dataset, outperforming existing methods (Jeribi et al., 2025).

An XAI-based smart agriculture framework was proposed for precision farming by integrating optimized feature selection using the Enhanced Barnacles Mating Optimization (EBMO) algorithm with machine learning models. The XLNet+SVM model achieved superior crop yield prediction accuracy, while SHAP and LIME enhanced the interpretability and transparency of AI-driven recommendations for sustainable agriculture (Martin et al., 2024).

The following are the major contributions of this study:

- Development of an intelligent crop recommendation framework based on Tab Transformer, Krill Herd Optimization (KHO), and Explainable Artificial Intelligence (XAI) techniques.
- Optimization of feature learning and hyperparameters to enhance the predictive performance of the proposed framework.
- Application of XAI techniques, including SHAP and LIME, to interpret model predictions and provide transparent decision-making support.
- Comprehensive evaluation of the proposed framework using multiple performance metrics to validate prediction accuracy and reliability.
- Development of a trusted and practical decision-support system for precision agriculture applications.

The remainder section of this paper is structured as follows: Section 2 reviews related work on crop recommendation & prediction using smart agriculture Section 3 presents a detailed overview of the materials and methods. Section 4 presents the experimental setup. Section 5 proposed model and compared with results and discussion of the model. Section 6 conclusion of the paper with contribution and limitations finally Section 7 focussed on future work.

## 2 Related work

This paper explores some of the challenges in providing AI-driven cropping advice and how this can be achieved with the help of XAI methods, such as LIME, SHAP, IG and LRP, to increase the reliability of the cropping advice. The purpose of integrating AI and XAI is to provide reliable advice on sustainable agriculture and food security (Akkem et al., 2024). The study introduces a deep ensemble model (CNN, CNN-SVM, DNN) to classify four potato leaf diseases with the accuracy of 99.98% and gives the introduction of LIME and SHAP to provide interpretability and trust (Paul et al., 2024). It is found that visualization techniques are most widely used, followed by rule-based system and feature selection, in the process of reviewing the applications of XAI in social media, healthcare and finance. It demonstrated, based on analysis of 70 + studies, that XAI increases trust, enhances model accuracy and interpretability (Malwade and Budhavale, 2023). XAI algorithms for early crop classification and identification of key stages of growth phases are employed here. For self-attention mechanisms, they improve the explainability of this process, the patterns in the crop development (Murindanyi et al., 2024). XAI Fruit Net outperforms state-of-the-art models with more than 97% accuracy and with good generalization, it is the first model to increase the transparency, trust, and achieve a new standard in explainable AI for the agriculture field (Sultana et al., 2024). SHAP and LIME are prominent XAI methods for providing explanations for model predictions. LIME and SHAP have been compared to illustrate the variable contribution and feature importance, respectively, by using local linear approximations and game theory-based Shapley values (Ishikawa et al., 2023). Random Forest is better in disease forecasting with 87% accuracy and was further improved to 90% after fine-tuning, whereas SVM and Naive Bayes had lower accuracy rates. This reveals the potential of AI and the need for optimisation of AI models (Abbas et al., 2024). In this study, DenseNet201 is employed to extract features, SVM to classify the features, and LIME to interpret those features in plant disease detection at an early stage. The system is transparent and helps farmers to take timely action (Ethiraj and Paranjothi, 2024). The study suggests a hybrid of the VGG19 model and the CNN model for sunflower disease recognition, which is better than eight models, namely precision, recall, F1 score, and accuracy, and is able to classify four types of sunflower diseases from a limited database (Ghosh et al., 2023). Author proposes a hybrid VGG19 + CNN model for sunflower disease detection which successfully classified four types of disease from a small-sized dataset with better precision, recall, F1 Score and accuracy than eight models (Naga Srinivasu et al., 2024). This study was conducted to extract the important soil properties for evaluating MARS, KRR, AdaBoost and MBA-Cat Boost models from 22 soil properties at 8 sites in Atlantic Canada, with the aid of SHAP, AdaBoost, MBA and Select Best to optimize hyperparameters (Jamei et al., 2024). XAI enhances the explainability of ML models while maintaining high accuracy. Techniques are divided into three categories; depending on whether they are model selection or post-hoc, model generality, and explanation scale (Ryo, 2022). The Optimally Heterogeneous Irrigation (OHI) system utilizes WSNs to estimate the requirements for irrigation water based on soil and crop conditions, thereby increasing the water efficiency, yield and cost-effectiveness under constraints of irrigation usage (Hamouda and Phillips, 2019). The novel KHbCN framework is based on CNN and an improved version of krill herd fitness function is used to achieve accurate detection of crop diseases from the data set Plant Village, using noise-free texture features for improving classification and enabling early intervention in the case of crop disease detection (Parthiban et al., 2023). However, there are limitations in crop disease detection such as low accuracy and overfitting. To address this, a novel KHbRF model is proposed to improve the accuracy and effectiveness of the detection process by adding an optimized krill herd fitness function to the Random Forest classifier (Srinivas et al., 2024). In this research, novel MHKHA is introduced to enhance the KHA by swap mutation and feature selection using k-means clustering. Seven benchmark datasets are used to test MHKHA and it is found that MHKHA performs better than other optimization methods (Abualigah et al., 2021). The ANFIS-KHA model combines ANFIS and Krill Herd Algorithm to enhance soil moisture estimation, reducing the RMSE by 28% to 1.94 RMSE and 0.05 MAPE, thereby improving the model’s accuracy (Moazenzadeh et al., 2022). Using the Stud Krill Herd Algorithm (SKHA) for optimal DG placement in radial system to minimize the power loss and cost, improve the voltage stability and accelerate the convergence in IEEE 33, 69, and 94 bus systems (Chithra Devi et al., 2021). The authors in this study suggest using XAI and blockchain technology in smart agriculture to enhance food safety, transparency in food supply chains, and decision-making. The research trends are identified by bibliometric analysis and the potential of these technologies are highlighted to develop sustainable and reliable agricultural systems (Chen et al., 2023). This study aims to establish the AI-based Smart Agriculture Prediction System combining with intelligent agent’s soil classification, soil parameter estimation, crop recommendation and fertilizer recommendation. The system utilizes high accuracy of soil classification (92.88%), crop recommendation (92.4%) and fertilizer recommendation (94.7%) with the use of deep learning and machine learning models. Moreover, the Explainable AI methods like SHAP and LIME are included to boost transparency, trust, and decision-making in precision agriculture (Swati et al., 2026). This paper delves into the use of AI, IoT, machine learning, remote sensing, and Variable Rate Technology (VRT) in smart agriculture and how they contribute to improved agricultural productivity and sustainability. The authors emphasize the applications of crop mapping, crop recommendation, disease detection, soil condition prediction and water stress monitoring. The study highlights the potential of AI-driven technologies in precision agriculture for efficient use of resources and decision-making (Pöchtrager et al., 2025). Smart Farming Technologies (SFTs) are digital and data-driven technologies that increase the productivity and sustainability of agriculture. But farmers are facing difficulties like high cost, privacy concerns, training and integration issues with the systems. The study emphasizes the necessity of providing more advanced decision support, data security, and knowledge transfer from technology providers to the society to speed up the implementation of smart farming practices and implement Agriculture 5.0 (Tariq et al., 2025). This article introduces a Smart Agriculture framework based on edges, which is suitable for connectivity constrained, energy constrained environments. Combining lightweight deep learning and rule-based AI on IoT devices allows for real-time weather classification on device without the need for a cloud server (Motamedi and Villányi, 2024). Despite having 19.9% share in the GDP, agriculture is still underutilizing the modern approach. Through this study, the author proposes the use of ML classifiers (SVM, KNN, DT, LR) combined with voting/stacking ensemble techniques which achieved the accuracy of 99.32% in crop recommendation using Moth Flame Optimization (MFO) (Choudhury et al., 2023). This research proposes an integrated intelligent agriculture system to solve the issues of soil classification, crop recommendation, and fertilizer recommendation. The proposed system uses a Custom CNN for soil classification with an accuracy of 92.88%, Random Forest for crop recommendation with an accuracy of 92.4% and XGBoost for fertilizer recommendation with an accuracy of 94.7%. Moreover, SHAP and LIME are included to make predictions transparent and reliable, which enhances the process of decision making in precision agriculture (Goel et al., 2026). The aim of this study is to present a machine learning framework for crop recommendation based on the climate and soil data from 3,961 cities in Brazil. Both the combined climate and soil information and Random Forest gave the best result, while Gradient Boosting gave the best city-specific F1-score of 0.70. The results show that the localized crop recommendation models deliver better prediction accuracy than the generalized models, which can enable better practices in precision agriculture (Gomes da Costa Cavalcanti et al., 2026). The objective of this study is assessing five ensemble learning algorithms (Random Forest, Extra Trees, CatBoost, XGBoost and LightGBM) for crop recommendation using soil and environmental parameters. The models had an accuracy of more than 98%, and Random Forest had the most stable accuracy. Ensemble learning proved to be effective in achieving accurate and sustainable crop recommendation, with the most important factors identified to be “rainfall” and “humidity” through feature importance analysis (Marlina et al., 2026). In this article, a crop recommendation system has been proposed using the Internet of Things (IoT) technology that integrates real-time soil and weather data received from the LoRa-based sensors for the accurate prediction of crops. The system is deployed in cloud environment using XGBoost algorithm which surpasses the limitations of traditional recommendation models, and the prediction accuracy of the system is 99%, which is higher than Decision Tree, Random Forest and ANN models. The framework offers a scalable and reliable decision support solution in precision agriculture (Balaji et al., 2026). Machine Learning-based dual-module agricultural decision support system for crop and fertilizer recommendation is presented in this study. The best results were obtained for the Random Forest model, with an accuracy of 99.32% for the crop recommendation and 98.75% for the fertilizer recommendation. Combining SMOTE, Grid Search CV and SHAP, the framework ensures a more accurate prediction, optimization of the model and a more transparent explanation of its operation, which is very useful in precision agriculture (Bülbül et al., 2026). The crop recommendation model is proposed in this study, which predicts appropriate crops based on soil nutrients and environmental parameters, by employing Gradient Boosting. The model achieved 99.27% accuracy, 99.32% precision, 99.36% recall, and a 99.32% F1-score. Explainable AI (XAI) is integrated to improve transparency by making sense of recommendations for precision agriculture (Shastri et al., 2025). Agrico-Net is proposed as a hybrid framework integrating CNN, LSTM, and the Whale Optimization Algorithm (WOA) for IoT-enabled precision agriculture. The model analyzes spatiotemporal sensor data to monitor crop health and optimize irrigation and fertilizer application, achieving 98.54% classification accuracy while significantly improving water-use efficiency, reducing fertilizer consumption, and enhancing agricultural sustainability (Ramu et al., 2026). To address the challenge of predicting the yield of corn and wheat, this paper presents the hybrid model of CNN, LSTM, and Whale Optimization Algorithm (WOA) for IoT-based precision agriculture, which is named as AgriCLWO-Net. The model is used to monitor crop health by relying on the spatiotemporal data gathered by the sensors in order to optimize watering and fertilizing of the crops. It was able to classify with 98.54% accuracy, has a high-water use efficiency, a lower fertilizer uses and a better agricultural sustainability (Ennaji et al., 2026). AgroSense is introduced as a multimodal deep learning framework that integrates soil image classification and nutrient profiling for real-time crop recommendation. The framework combines CNN- and Transformer-based soil classification with machine learning models for structured soil data analysis, achieving 98.0% accuracy and demonstrating an effective, scalable solution for precision agriculture with statistically validated performance improvements (Pandey et al., 2025).

Table 1 presents the State-of-the-Art Comparison (SoA) analysis of recent studies on AI-based crop recommendation and classification systems. The comparison highlights the application domains, machine learning models, explainable AI techniques, optimization methods, and key limitations, thereby identifying the research gap addressed by the proposed framework.

**Table 1:** State-of-the-art comparison of existing studies in explainable AI and smart agriculture.

References	Year	Application area	AI/ML model	XAI technique	Optimization method	Limitation
Parthiban et al. (2023)	2024	Crop recommendation	AI-based recommendation	LIME, SHAP, IG, LRP	No	Focused on explainability; no optimization framework
Srinivas et al. (2024)	2023	Potato disease classification	CNN, CNN-SVM, DNN	LIME, SHAP	No	Limited to disease detection
Moazenzadeh et al. (2022)	2024	Crop classification	Self-attention model	XAI methods	No	No optimization strategy
Chithra Devi et al. (2021)	2024	Fruit classification	XAI FruitNet	Explainable framework	No	Focused on fruit recognition only
Swati et al. (2026)	2024	Disease forecasting	Random Forest, SVM, Naïve Bayes	No	Fine-tuning	Moderate prediction accuracy
Pöchtrager et al. (2025)	2023	Plant disease detection	DenseNet201 + SVM	LIME	No	Disease-specific application
Tariq et al. (2025)	2023	Sunflower disease detection	VGG19 + CNN	No	No	Small dataset used
Choudhury et al. (2023)	2024	Soil property prediction	MARS, KRR, AdaBoost, CatBoost	SHAP	MBA Optimization	Limited focus on soil analysis
Marlina et al. (2026)	2023	Crop disease detection	CNN	No	Krill Herd Algorithm (KHA)	No explainability support
Balaji et al. (2026)	2024	Crop disease detection	Random Forest	No	KHA Optimization	Lack of model interpretability
Bülbül et al. (2026)	2021	Feature selection optimization	Machine Learning	No	Modified KHA	Not applied to crop recommendation
Shastri et al. (2025)	2022	Soil moisture estimation	ANFIS	No	KHA	Focused on estimation rather than recommendation
Ennaji et al. (2026)	2023	Smart agriculture	AI + Blockchain	XAI	No	Conceptual framework
Pandey et al. (2025)	2026	Crop and fertilizer recommendation	Deep Learning + ML	SHAP, LIME	No	No optimization-based tuning
Proposed Work	2026	Crop recommendation	MLP, XGBoost, TabTransformer	SHAP, LIME	KHO, GA, PSO, SA	Addresses both interpretability and optimization

### 2.1 Research gap analysis

The use of AI and ML has been shown to be effective in crop recommendations, disease detection, and smart agriculture applications, among other areas. There are several works that use explainable AI techniques like SHAP and LIME to make the AI more transparent, and other works that use optimization algorithms to make the AI more accurate. Most studies however focus either on explainability or optimization separately. The integration of multiple advanced prediction models, explainable AI methodologies and several metaheuristic optimization algorithms to crop recommendation is still not well explored. To fill the gap, the proposed work combines MLP, XGBoost and Tab Transformer models are integrated with SHAP, LIME, KHO, GA, PSO and SA to provide accurate, interpretable and optimized recommendations for crop selection in precision agriculture.

## 3 Materials and methods

This section introduces the methodology used for the recommended scheme of crops. The framework integrates soil nutrient information, climatic variables, transformer-based learning, Explainable Artificial Intelligence (XAI) and Krill Herd Optimization (KHO) to boost predictive accuracy of crop yields and their model interpretability. The methodology involves five consecutive steps: (1) designing the framework, (2) designing the integrated nutrient–climate feature learning, (3) crop prediction using the transformer, (4) hyperparameter optimization using the KHO, and (5) explainability analysis with SHAP and LIME for making reliable agricultural decisions in sustainable farming practices.

### 3.1 Proposed crop recommendation framework

**Figure 1:**
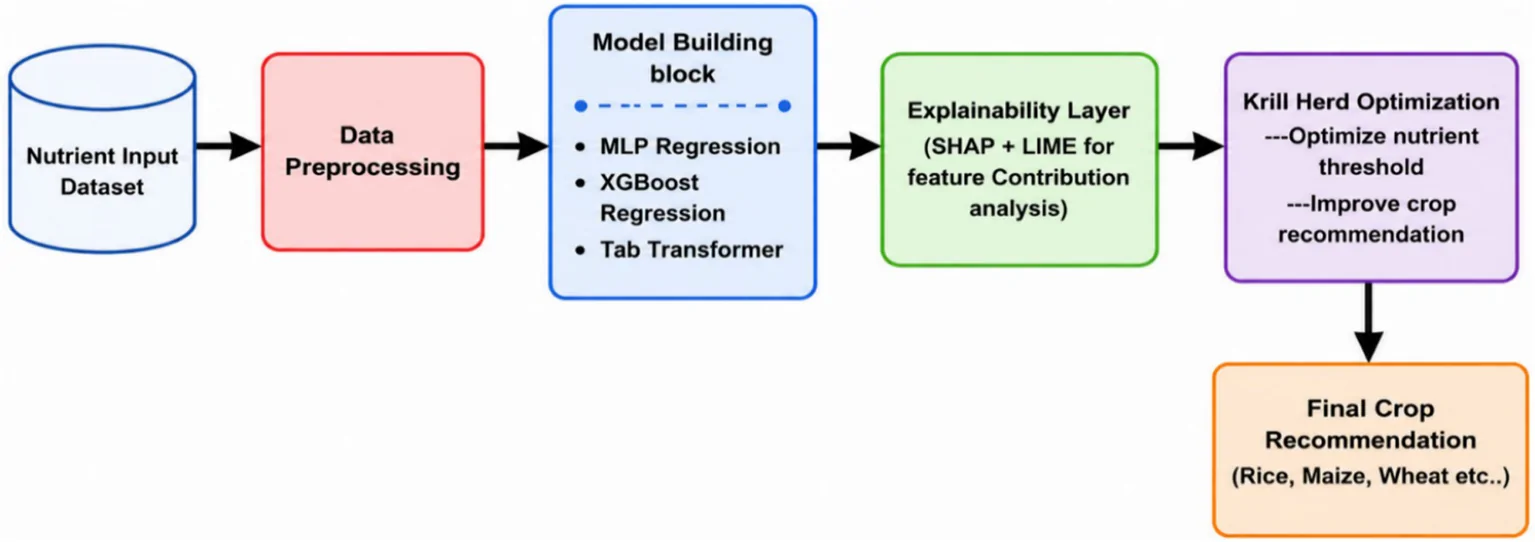
Overall workflow of the proposed transformer-based crop recommendation framework integrating KHO optimization and XAI.

Overall architecture of the proposed crop recommendation framework is shown in Figure 1. The system integrates Tab Transformer, a crop forecasting model, with Explainable Artificial Intelligence (XAI) for interpreting the model results and Krill Herd Optimization (KHO) for hyperparameter tuning. The proposed approach can be broken down into four subsequent steps: (i) Soil nutrient and climatic data preprocessing, (ii) Crop prediction using transformer networks, (iii) Hyperparameter optimization using KHO, and (iv) Explainable decision support. The framework starts with the collection of soil nutrient properties like nitrogen (N), phosphorus (P), potassium (K), copper (Cu), iron (Fe), magnesium (Mg), sulphur (S) and climatic attributes like temperature, humidity, pH and rainfall. After pre-processing, these integrated nutrient–climate features are fed into the Tab Transformer model that learns complex feature interactions to make the best possible prediction for the most appropriate crop for a specific agricultural context. Further, KHO is employed to fine-tune model hyper-parameters to improve the predictive performance and thereby better classifications of crops. Lastly, SHAP and LIME are applied to provide global and local explanations of the prediction process, thus explaining the role of each nutrient and climatic feature in each recommendation. By combining the concepts of transformer-based learning, optimization, and explainable AI, precision agriculture can benefit from a reliable, transparent, and accurate framework to make crop recommendations.

### 3.2 Integrated nutrient–climate feature learning framework

The proposed framework for integrating some of the nutrients and climate is a mixture of soil nutrient and environmental features which can be able to integrate the interaction of these features and make an intelligent recommendation for crop. The input factors involved are macronutrients (N, P, K) micronutrients (Cu, Fe, Mg, S) and climatic factors (temperature, humidity, pH, rainfall). By combining these complementary attributes, the model can learn more comprehensive representations of soil fertility and soil environmental conditions that affect suitability of crops.

The attention mechanism-based Tab Transformer model takes the integrated feature set as input to learn complex relationships among nutrient and climatic variables for the prediction of crops. In order to improve the prediction accuracy, Krill Herd Optimization (KHO) is used to optimize the model hyperparameters. Later, explanations are provided to the prediction results, using SHAP and LIME, which provides both global and local explanations, by identifying the contribution of each feature among the recommendations for each crop. A comprehensive and explainable framework is established for precise crop recommendations in various agricultural contexts through the integration of feature learning, optimization, and explainability.

### 3.3 Transformer-based crop prediction model

The framework for crop recommendation proposed is Tab Transformer model which learns complex relationships between soil nutrient and climatic features to make most suitable crop recommendation. The transformer architecture does not have the same structure as the traditional machine learning models as it uses the self-attention mechanism to capture the interactions between the agricultural attributes, effectively representing tabular data and predicting crop outcomes. Integrated nutrient–climate features, such as macronutrients (N, P, and K), micronutrients (Cu, Fe, Mg, and S), and environmental factors (temperature, humidity, pH, and rainfall) are fed to the model. Such input features are then embedded to represent them and passed to self-attention layers for knowledge acquisition about the relationship between the nutrient and climatic variables. The obtained feature representations are then supplied to the predicting layer where the most suitable crop is predicted under the given agricultural conditions. The transformer model’s feature learning offers a valuable ability to capture key interactions among soil nutrients and environmental conditions, resulting in more robust predictions and accurate classification.

The optimized prediction model is then further enhanced with hyperparameter optimization using KHO algorithm and interpretable explanation of the crop recommendation process is provided using SHAP and LIME.

### 3.4 KHO-driven hyperparameter optimization

The hyperparameters of Tab Transformer model are optimized using a method called Krill Herd Optimization (KHO), and the prediction accuracy of the crop is improved. A swarm-based optimization algorithm, iterative for the best set of model parameters, KHO evaluates different possible solutions and iteratively updates them towards the optimum fitness. This optimization not only enhances the prediction model’s learning performance, but also minimizes classification errors. The optimized hyperparameters are then used to train the final Tab Transformer model, demonstrating better prediction accuracy, robustness and generalization across various agricultural environments. Optimized prediction model is then used to perform explainability analysis with SHAP and LIME to offer interpretable and transparent crop recommendations.

### 3.5 Explainable AI-based decision support

The proposed framework incorporates Explainable Artificial Intelligence (XAI) techniques to enhance transparency and interpretability of crop recommendations. SHAP is used for quantifying the impact of nutrient and climatic features on the prediction, by giving global feature importance and instance-specific explanation. LIME is to complement the SHAP by providing local explanations of the individual crop recommendations, thus allowing users to gain insight into the factors behind each prediction. The combination of SHAP and LIME helps to make the proposed crop recommendation framework interpretable, as it can offer valuable insights into the prediction process. These explanations increase the confidence in the model and enable making better decisions, making it more appropriate for the use in practical precision agriculture.

#### 3.5.1 SHAP (SHapley Additive Explanations)

SHAP (SHapley Additive Explanations) is used to explain the predictions of the proposed crop recommendation system by measuring the contribution of each feature in the input to the output of the model. SHAP determines the importance value of each nutrient and climatic attribute based on game theory, which is related to the impact on the predicted crop. It gives both world-wide and local justification, which allows the most important soil nutrient and environmental factors contributing to crop recommendation to be identified. The feature importance analysis that results from this makes the proposed framework more transparent and reliable.

#### 3.5.2 LIME (local interpretable model-agnostic explanations)

LIME (Local Interpretable Model-agnostic Explanations) is used to give local explanations to the individual crop recommendations produced by the proposed framework. By approximating the prediction of the Tab Transformer model with a simple interpretable model, it identifies the contribution of nutrient and climatic features for a particular prediction. This allows users to see the effect of each individual variable in the input on the recommended crop, thus adding the transparency and interpretability of the decision-making process.

## 4 Experiment setup

The experimental setup was set up to test the effectiveness of the proposed framework for providing explanations of crop recommendations. The experiments consisted of data set preparation, feature preprocessing, model training, hyperparameter optimization, and model performance evaluation. To achieve a fair evaluation, several machine learning and deep learning models were designed and tested in the same experimental environment. Computational environment, characteristics of the data, preprocessing processes, validation method, and evaluation results are explained in the following subsections.

### 4.1 Computational environment

The proposed crop recommendation framework was implemented using a Python-based computational environment. An experimental setup was built, which comprised both the hardware and the software environment. The hardware consisted of an Intel Core i7 processor, 16 GB of memory, and a computational and deep learning accelerator, NVIDIA GPU. Python 3.10 (Jupyter Notebook) was used for the software environment, supplemented by packages such as NumPy, Pandas, Scikit-learn & XGBoost, TensorFlow/Keras, SHAP and LIME for machine learning and model interpretability, and Matplotlib & Seaborn for visualization & performance analysis.

### 4.2 Dataset description

The dataset used in this study was obtained from the publicly available Kaggle crop recommender dataset with soil nutrients (Kaggle, 2021).

Crop Prediction for Sustainable Farming with Composition of Minerals. The dataset consists of 2,200 samples with 11 input features representing soil nutrient and environmental parameters used for crop recommendation and agricultural decision-making. The input attributes include Nitrogen (N), Phosphorus (P), Potassium (K), Copper (Cu), Iron (Fe), Magnesium (Mg), Sulphur (S), Temperature, Humidity, pH, and Rainfall, while the target variable corresponds to crop categories used for prediction. Prior to model training, preprocessing operations were performed to improve data quality and reliability. Initially, the dataset was examined for inconsistencies and redundant information to ensure data integrity. Numerical features were normalized using Min–Max scaling to transform values into a common range and reduce variations caused by differences in feature magnitude. Crop labels were converted into numerical form using One-Hot Encoding to facilitate efficient model processing. The dataset was then divided into training and testing subsets using an 80:20 ratio for model development and evaluation. The preprocessing stage improved data consistency and enhanced the effectiveness of the proposed framework during training and prediction.

### 4.3 Data pre-processing

#### 4.3.1 Label encoding

The crop labels in the dataset were categorical in nature and therefore required conversion into numerical representations for machine learning model compatibility. Label encoding was employed to transform crop names into integer values while preserving unique class identification. This transformation enabled efficient processing by the classification algorithms.

#### 4.3.2 Handling missing values

Missing values in the dataset were handled using mean imputation. Any null values present in numerical attributes were replaced with the mean of the respective feature column. This approach helped maintain data consistency and prevented information loss that could occur from removing incomplete records.

#### 4.3.3 Feature normalization

Since the dataset contained features with different numerical ranges such as Nitrogen (N), Phosphorus (P), Potassium (K), temperature, humidity, pH, and rainfall, normalization was performed to scale the values into a common range. This process reduced the impact of feature magnitude differences and improved model performance and convergence.

#### 4.3.4 Feature selection

Relevant input features including soil nutrients (N, P, K), environmental parameters (temperature, humidity, rainfall), and soil characteristics (pH) were selected for crop recommendation analysis. Feature selection was performed to eliminate irrelevant data, reduce dimensionality, improve computational efficiency, and enhance prediction accuracy.

#### 4.3.5 Data splitting and validation strategy

The pre-processed data was split into an 80:20 training and testing set, with 80% of the data used to train the model, and 20% used to finalise the results of the model’s performance. Stratified data splitting approach was used to ensure the original distribution of the crop classes in the training and testing sets. The model was further validated using the training dataset in the optimization of the model parameters. This approach provided a reliable evaluation of the models and minimized the likelihood of a biased model performance estimate.

#### 4.3.6 Model training and parameter settings

The models were trained with the pre-processed dataset using the right hyperparameter set for the selected machine learning and deep learning models. These parameters depend on the model, such as learning rate, batch size, epochs, configurations of algorithm etc., and were set prior to training. For machine learning models, parameters included number of estimators, tree depth, and for deep learning models, predefined training parameters were used for FT-Transformer, DNN, and 1D-CNN. The KHO algorithm was used for hyperparameter optimization to adjust the population size and number of iterations. The models were trained under identical experimental conditions to allow reliable comparisons of model performance.

#### 4.3.7 Justification for algorithm selection

The selected algorithms were chosen based on their capability to effectively handle multidimensional agricultural datasets and identify complex relationships among soil properties and environmental conditions. The algorithms were also selected due to their prediction capability, computational efficiency, and suitability for crop recommendation tasks. Appropriate parameter settings were chosen to achieve a balance between model complexity, training time, and prediction accuracy.

### 4.4 Algorithm: proposed explainable crop recommendation framework

#### 4.4.1 Algorithm description

The proposed framework consists of two stages: baseline model development and enhanced crop recommendation. Initially, the baseline models, namely Multilayer Perceptron (MLP) and Extreme Gradient Boosting (XGBoost), are trained to establish benchmark prediction performance. Subsequently, the proposed enhanced model employs a Tab Transformer optimized using Krill Herd Optimization (KHO) to improve feature representation and prediction accuracy. Finally, the predictions generated by the baseline and enhanced models are combined through ensemble averaging, while SHAP and LIME are utilized to provide global and local interpretability, resulting in accurate, reliable, and explainable crop recommendations.

ALGORITHM 1 Proposed explainable crop recommendation framework.

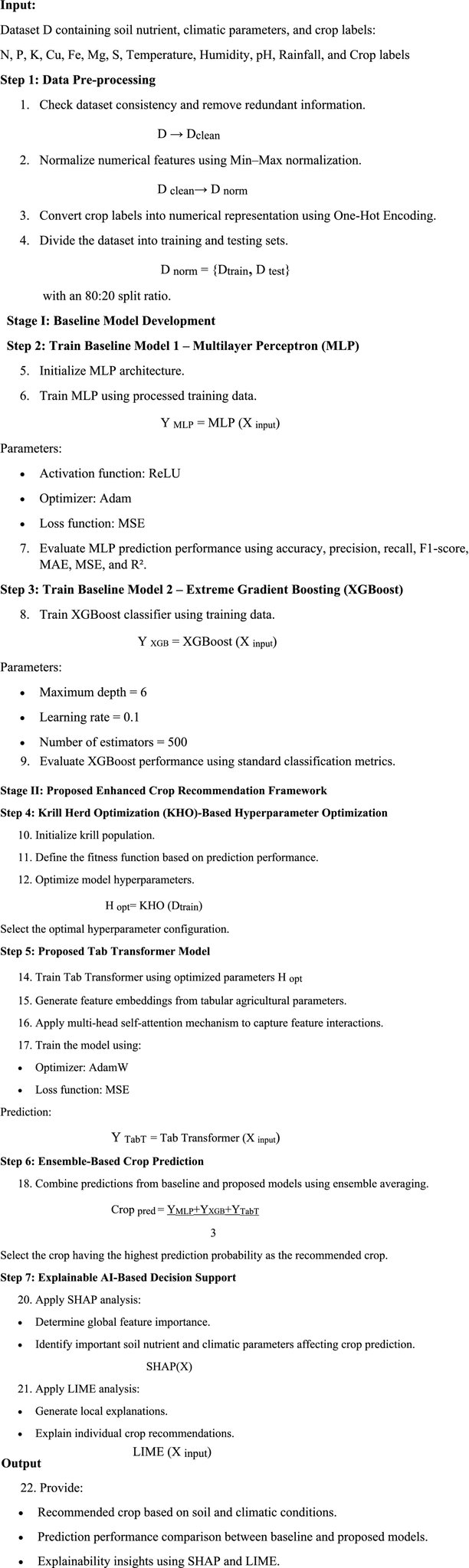

### 4.5 Evaluation metrics

The performance of the proposed crop recommendation framework is evaluated using both classification and regression metrics. For classification, Accuracy, Precision, Recall, and F1-Score are employed to assess the effectiveness of crop prediction. For regression analysis, Mean Absolute Error (MAE), Mean Squared Error (MSE), and the Coefficient of Determination (R^2^) are used to evaluate prediction errors and model fit as shown in Equations 1–7.


$$\text{Accuracy}=\frac{\mathrm{TP}+\mathrm{TN}}{\mathrm{TP}+\mathrm{FN}+\mathrm{FP}+\mathrm{TN}}$$ (1)

$$\text{Precision}=\frac{\mathrm{TP}}{\;\mathrm{TP}+\mathrm{FP}}$$ (2)

$$\text{Recall}=\frac{\mathrm{TP}}{\;\;\mathrm{TP}+\mathrm{FN}}$$ (3)

$$F1-\text{Score}=2*\frac{\text{Precision}*\text{recall}}{\text{Precision}+\text{recall}}$$ (4)

$$\mathrm{MAE}=\frac{1}{n}\sum_{i=1}^{n}|(y_{i}-\hat{Y}i)$$ (5)

$$\mathrm{MSE}=\frac{1}{n}\sum_{i=1}^{n}{\left(y_{i}-{\hat{Y}}_{i}\right)}^{2}$$ (6)

$$\;\;R^{2}=1-\frac{\sum_{i=1}^{1}(Y_{i}-{\hat{Y}}_{i})2}{\sum_{i=1}^{1}(Y_{i}-{\hat{Y}}_{i})2}$$ (7)

Where (TP), (TN), (FP), and (FN) represent true positives, true negatives, false positives, and false negatives, respectively. (Yi) denotes the actual value, (Ŷi) represents the predicted value, and ( $\hat{Y}$ ) indicates the mean of the actual values. MAE measures the average absolute prediction error, MSE emphasizes larger prediction errors through squaring, and R^2^ evaluates the proportion of variance explained by the model.

## 5 Results and discussion

This section presents the experimental evaluation of the proposed explainable crop recommendation framework. The performance of baseline and proposed models is analyzed using classification and regression metrics. Further, feature importance analysis, explainable AI interpretation, and KHO-based optimization results are discussed to demonstrate the reliability and interpretability of the framework.

### 5.1 Performance evaluation of baseline models

#### 5.1.1 Classification performance comparison of baseline and transformer models

**Figure 2:**
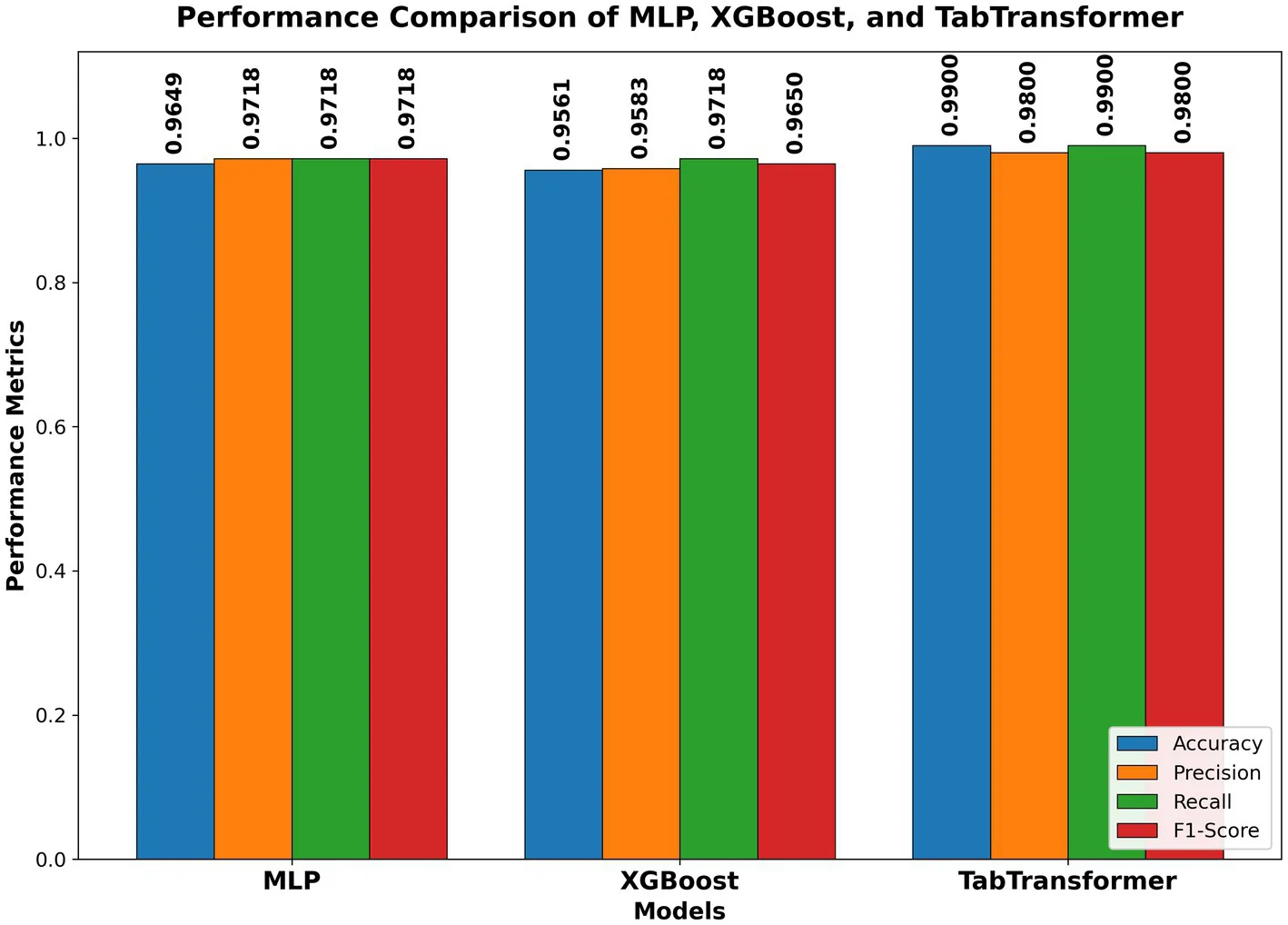
Performance comparison of MLP, XGBoost, and Tab Transformer.

The crop recommendation classification performance of MLP, XGBoost and Tab Transformer is shown in Figure 2. The results show that Tab Transformer consistently outperforms others in all the evaluation metrics. This improvement is due to its capacity to model complex interactions between soil nutrients and environment through an attention-based learning mechanism. Compared to this, the performance of MLP and XGBoost is relatively low, as they have less ability to model the relationship between contextual features in agricultural data.

Table 2 compares the classification performance of baseline models (MLP and XGBoost) with the Transformer-based model. MLP achieved an accuracy of 96.49%, while XGBoost obtained 95.61%, showing their capability to learn patterns from agricultural features. However, their performance was limited in capturing complex relationships among nutrient and climatic variables. The Tab Transformer achieved the highest performance with 99% accuracy, 98% precision, 99% recall, and 98% F1-score due to its self-attention mechanism, which effectively learns complex feature interactions in tabular agricultural data. These results indicate that Tab Transformer provides better crop classification performance and was selected as the base model for further KHO optimization and XAI analysis.

**Table 2:** Classification performance comparison of crop recommendation models.

Model	Accuracy	Precision	Recall	F1-score
MLP	0.9649	0.9718	0.9718	0.9718
XGBoost	0.9561	…	…	0.9650
Tab transformer	0.9900	0.9800	0.9900	0.9800

#### 5.1.2 Regression error analysis of prediction models

The performance of regression is compared with the help of MAE, MSE and R^2^ Score in Table 3. Tab Transformer showed the best predictive capability with the lowest MAE (0.10) and MSE (0.01) and the highest R^2^ Score (0.98). On the other hand, MLP performed worst in terms of prediction error and had the lowest R^2^ Score, whereas XGBoost showed moderate performance. The findings show that the Tab Transformer indeed outperforms its peers in learning complex patterns from agricultural data, leading to more precise and dependable forecasts.

**Table 3:** Comparative analysis of regression model performance.

Metric	TabTransformer regression	MLP regression	XGBoost regression
MAE	0.1	14.56	8.57
MSE	0.01	569.04	243.07
R2 score	0.98	0.45	0.69

#### 5.1.3 Feature importance analysis before optimization

**Figure 3:**
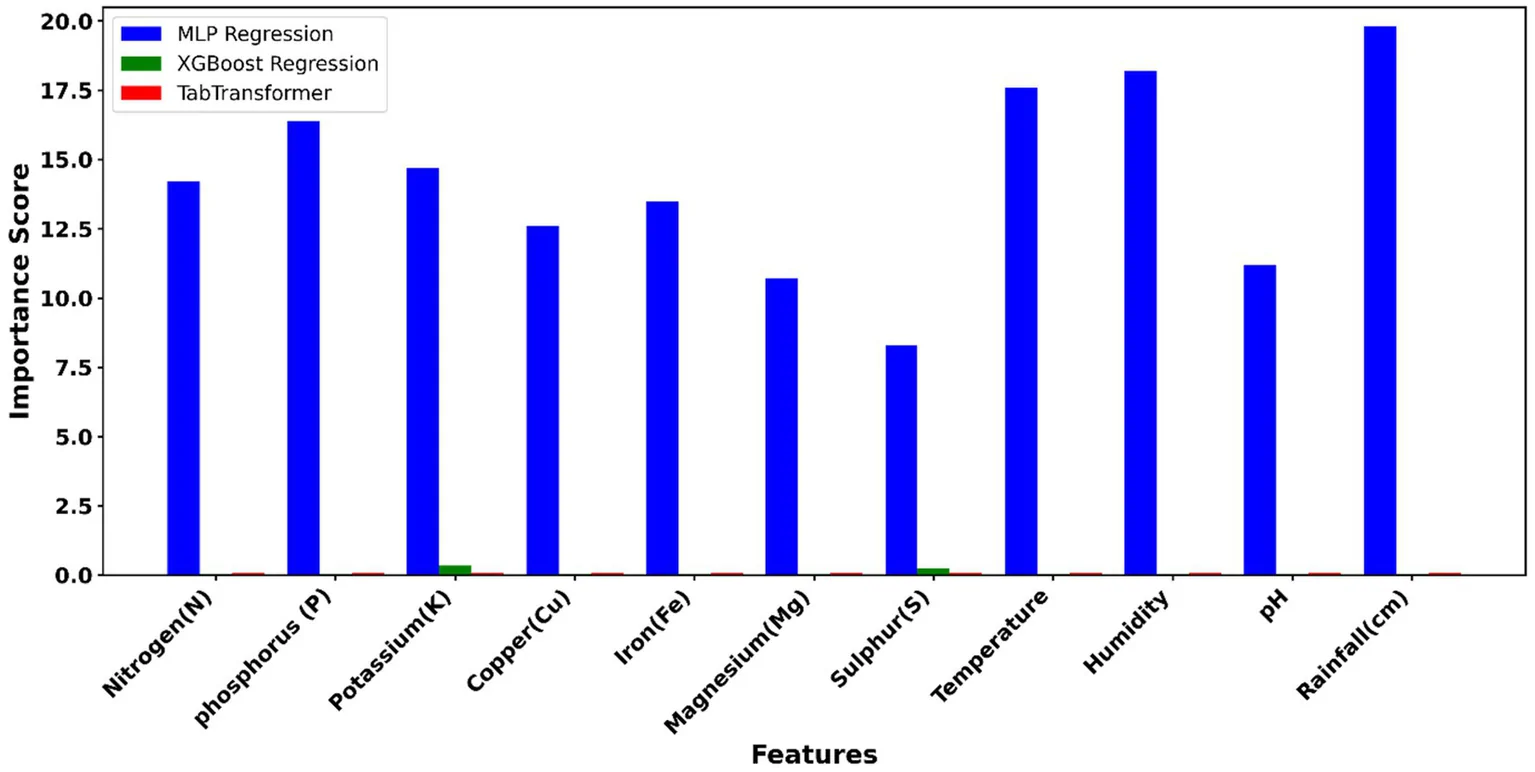
Feature importance comparison across MLP, XGBoost, and Tab Transformer models.

The feature importance analysis results in the model Figure 3 reveal the most important features for each model used to predict the crop type. The features analyzed are Nitrogen (N), Phosphorus (P), Potassium (K), Copper (Cu), Iron (Fe), Magnesium (Mg), Sulphur (S), temperature, humidity, pH and rainfall. Results show that the contribution of each feature is different for the models used based on their learning mechanism.

The most important feature for the MLP model is Phosphorus followed by Humidity, Rainfall and Potassium (K) indicating the importance of nutrients and environmental conditions in crop prediction. Potassium (K) and Sulphur (S) have the highest importance scores in the XGBoost model, suggesting that these elements play a significant role in the prediction process. Humidity, Phosphorus (P), Magnesium (Mg) also showed moderate effect and pH and Copper (Cu) showed comparatively lesser effect.

Figure 3 is a comparative analysis of the feature contribution for all the models. Nutrient related attributes are consistently important for crop prediction, but the importance of these features varies between the models, namely Phosphorus (P), Potassium (K), and Nitrogen (N). The suitability of crops is also influenced by environmental factors such as temperature, humidity and rainfall. The results indicate that the attribute meaning varies across different machine learning models, highlighting the need for explainable analysis of models to learn their behaviors and further assist in making reliable decisions on crop recommendations.

**Figure 4:**
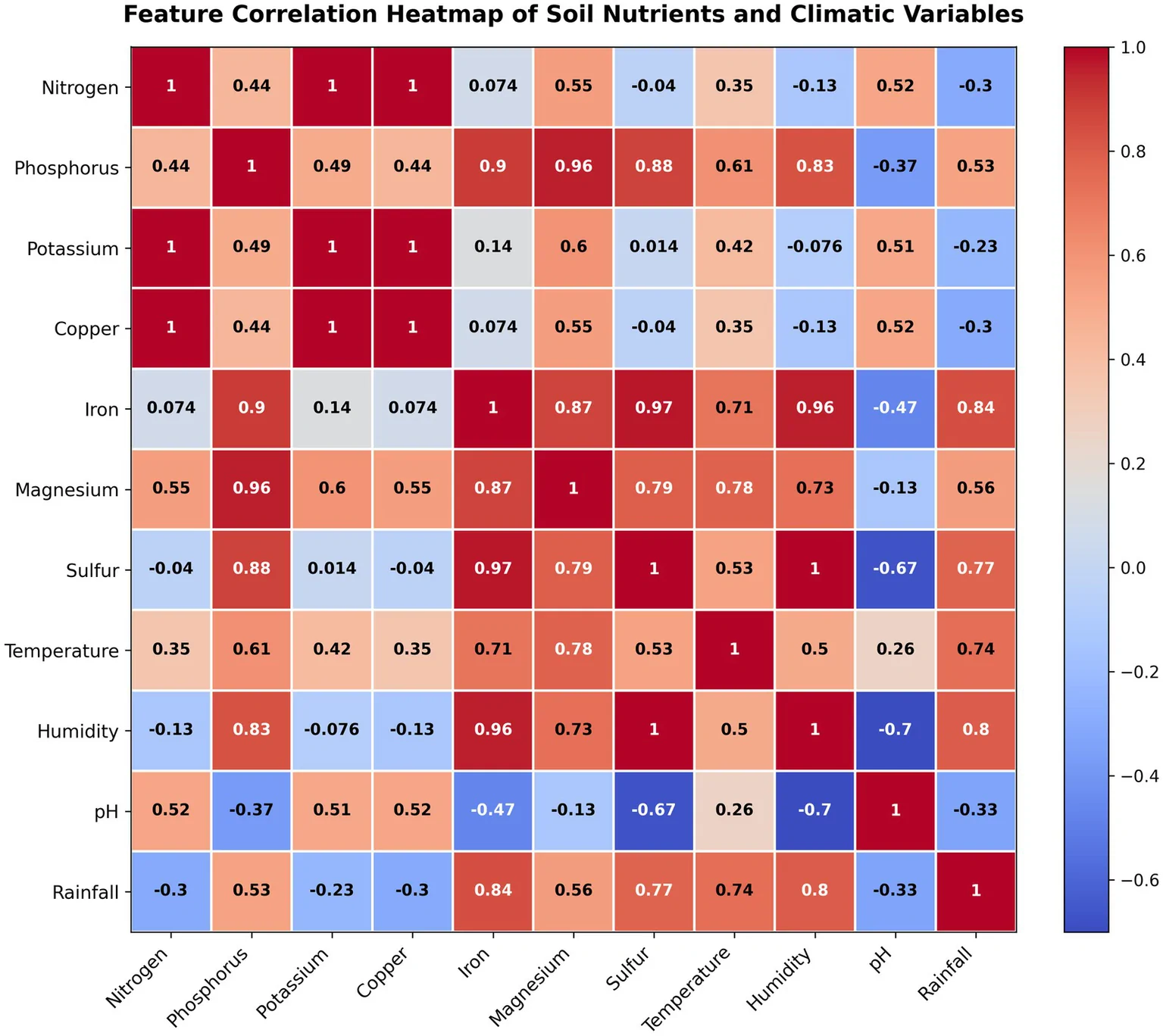
Feature correlation heatmap of soil nutrients and climatic variables.

The correlation heat map of the nutrient and environmental features used for crop prediction is presented in Figure 4. There are a number of positive relationships which are strong, such as Iron–Magnesium (0.97), Sulphur–Phosphorus (0.88) and Potassium–Phosphorus (−0.49), which show potential interactions between soil nutrients. There is a moderate positive correlation between humidity and rainfall (0.50), which indicates that they both influence the environment. Contrary to this, there is a negative correlation (−0.67) between pH and Sulphur, which indicates that there could be a possible influence of soil acidity on the availability of Sulphur. The heatmap gives a good insight of the relationships between the features and highlights the variables that are relevant for the correct crop recommendation.

### 5.2 Effect of nutrient and climate feature integration

To analyze the influence of different feature combinations on crop recommendation performance, experiments were conducted using only macronutrient features (N, P, K) and an integrated feature set consisting of macro nutrients, micronutrients, and climatic parameters. The objective of this analysis is to evaluate how additional soil and environmental information improves crop classification capability.

#### 5.2.1 Macronutrient-based prediction

The crop predictions made by the MLP, XGBoost and Tab Transformer models are provided in Table 4, when only the macronutrient features (Nitrogen (N), Phosphorus (P), and Potassium (K)) are used. Overall, the results show that the crop recommendation varies across the models, suggesting that macronutrient information alone may not be sufficient to have enough discriminatory power to classify the crops consistently. The observed differences emphasize the need of a soil’s primary nutrients as a single indicator of crop recommendation.

**Table 4:** Crop prediction based on macronutrient features.

Sample ID	Nitrogen (N)	Phosphorous (P)	Potassium (K)	Predicted crop (tab transformer)	Predicted crop (MLP)	Predicted crop (XGBoost)
1,451	101	17	47	Muskmelon	Watermelon	Watermelon
1,334	98	8	51	Watermelon	Watermelon	Watermelon
1761	59	62	49	Papaya	Banana	Papaya
1735	44	60	55	Papaya	Papaya	Blackgram
1,576	30	137	200	Apple	Apple	Coconut
1,110	18	19	27	Mango	Mango	Mango
94	35	145	195	Apple	Papaya	Grapes
530	22	44	24	Mothbeans	Mothbeans	Mothbeans
651	11	46	24	Mungbean	Mungbean	Mungbean
819	3	78	18	Lentil	Lentil	Mungbean

The results show that using only N, P, and K produces variations among model predictions, indicating that macronutrients alone may not provide sufficient information for accurate crop discrimination. This highlights the importance of including additional nutrient and environmental factors for improved crop recommendation.

#### 5.2.2 Integrated feature-based prediction

Table 5 is a comparative analysis of the crop prediction methods using only macro nutrients versus the combination of macro nutrients, micro nutrients and environmental factors. The comparison shows that adding further nutrient and climatic parameters gives more information for the recommendations of crop, which allows the better differentiation of crops and more comprehensive agricultural decisions. The macro nutrient-based approach provides generalized recommendations while the integrated feature approach provides more specific and context-aware crop prediction.

**Table 5:** Comparison of macronutrient and integrated feature-based approaches.

Aspect	Macro nutrient-based (first table)	Macro+ micro nutrient-based (second table)
Features used	N,P,K	N,P,K Copper, Iron, Magnesium, Sulfur, Temperature, Humidity, pH, Rainfall
Model performance	More generalized, some model disagreement	More refined, better crop differentiation
Prediction consistency	Higher disagreement across models	More adjustment across models due to additional features
Real-world applicability	Useful for general fertilizatin planning	More precise for crop recommendation and sustainability

Table 6 shows the predictions of the various crops made with the complete set of features comprising macro nutrients, micro nutrients (Copper, Iron, Magnesium and Sulphur), and environmental parameters (rainfall, temperature, humidity and pH). These extra characteristics are included to give a more complete picture of the agricultural situation and allow for more complex relationships to be represented which affect crop growth. The results show the impact of richer feature information on the crop prediction behavior and on making a better crop recommendation decision.

**Table 6:** Crop prediction based on nutrient and environmental features.

Sample ID	Nitrogen	Pkoshorous	Potassium	Copper	Iron	Magnesium	Sulphur	Temperature	Humidity	pH	Rainfall	Predicted crop (MLP)	Predicted crop (XGBOOST)	Predicted crop (tab transformer)
0	50	30	40	0.5	3	1.5	12	25	65	6.5	120	Rice	Coffee	Watermelon
1	80	40	60	0.8	4.5	2	15	30	70	6.8	140	Rice	Maize	Pigeonpeas
2	120	35	50	1.2	2.8	1.8	10	27	60	7	110	Watermelon	Coffee	Pigeonpeas
3	100	50	70	1	5	2.2	10	28	75	6.4	130	Rice	Rice	Pigeonpeas

### 5.3 Explainable AI analysis

Explainable Artificial Intelligence (XAI) techniques were applied to understand the decision-making process of the crop recommendation models. SHAP and LIME were used to identify important nutrient and climatic factors influencing crop predictions and to improve the transparency of the model decisions.

#### 5.3.1 SHAP summary analysis

**Figure 5:**
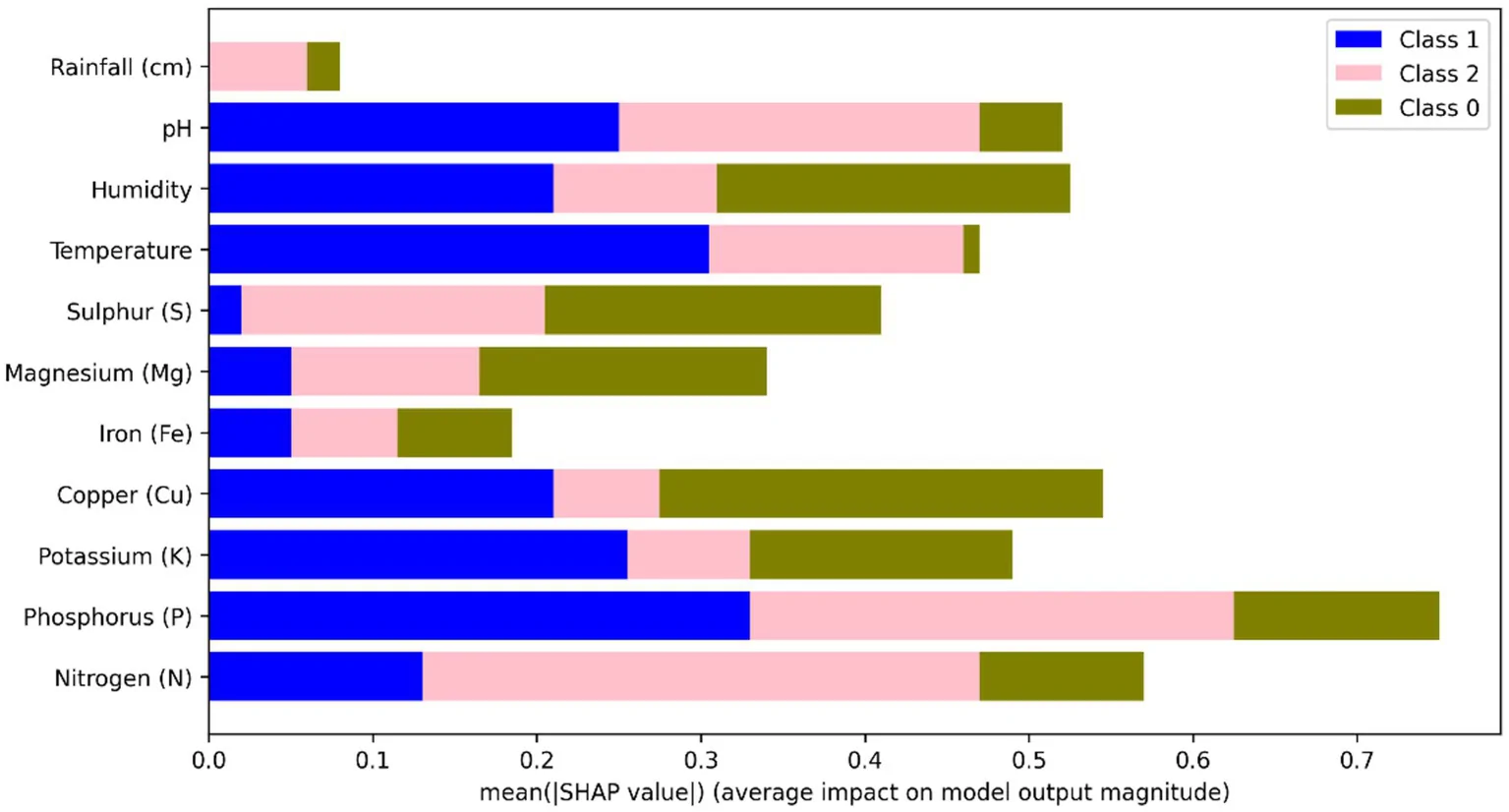
SHAP feature importance summary for crop recommendation model.

This SHAP (SHapley Additive Explanations) summary plot provides an interpretation of feature importance in the crop recommendation model, and shows how soil nutrients and climate factors affect predictions. The features (Nitrogen (N), Phosphorus (P), Potassium (K), Copper (Cu), Iron (Fe), Magnesium (Mg), Sulphur (S), Temperature, Humidity, pH, and Rainfall) are displayed along the y-axis in Figure 5, with the x-axis representing the mean absolute SHAP value, which measures the average impact of each feature on the model’s output. Crop classes (Class 1-blue, Class 2-pink, Class 0-olive green) are represented by color coding and represent each feature’s contribution per class. The top three features with the largest SHAP values are Nitrogen (N), Phosphorus (P), and Potassium (K), which are the most important features to consider in crop recommendations. There is also a significant class specific effect for Copper (Cu). The other features like Humidity, pH, Rainfall have moderate impact, and Magnesium, Iron, Sulphur have relatively low impact. This visualization allows for a better understanding and transparency of the model, which can aid in refining crop recommendations based on the data and key soil and environmental factors.

#### 5.3.2 SHAP decision analysis

**Figure 6:**
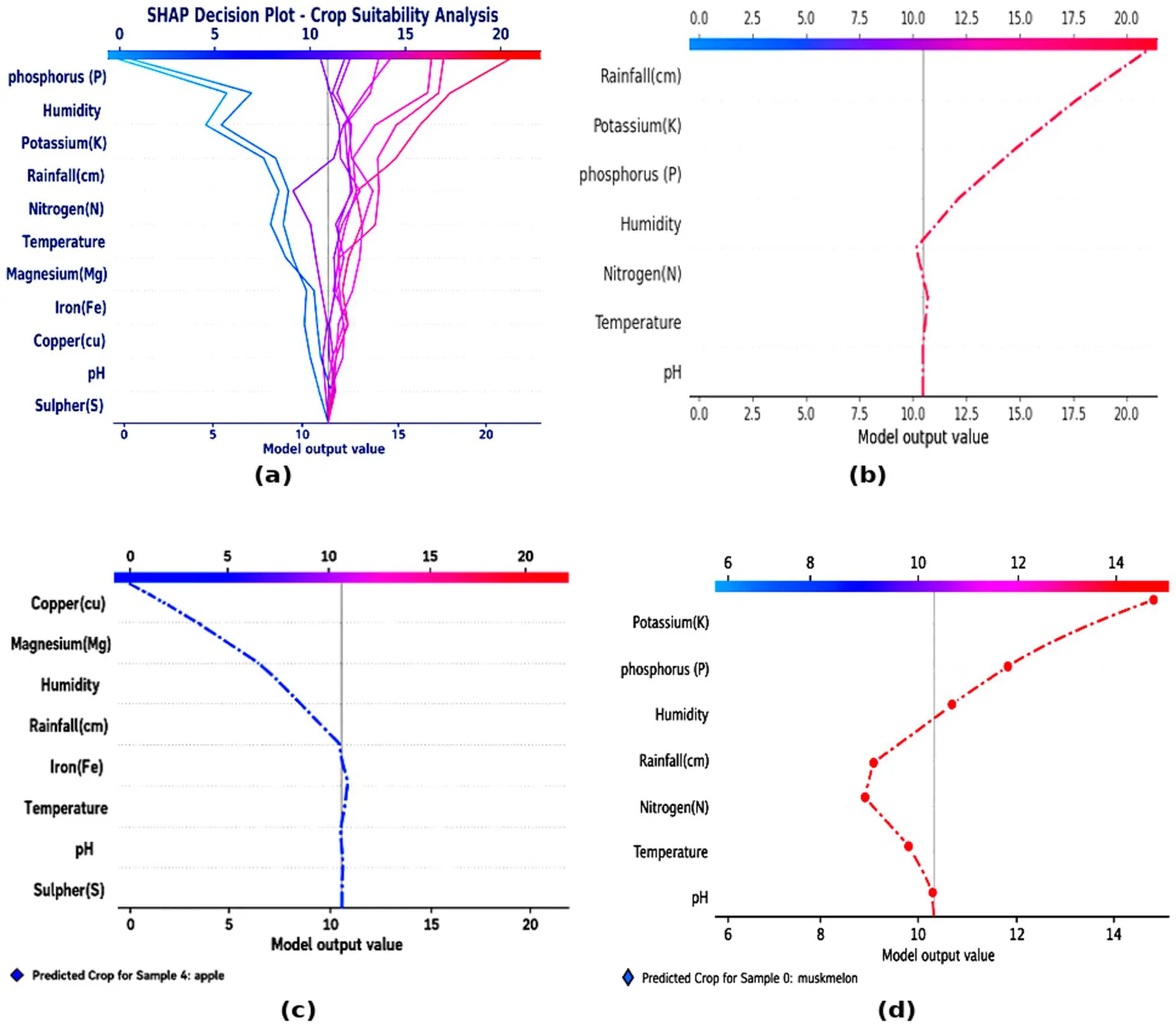
**(a)** SHAP-based contribution of individual soil and climatic features to the model output across crop classes. **(b)** Cumulative effect of the most influential features on the predicted model output. **(c)** Feature-wise contribution illustrating the direction and magnitude of each feature’s influence on crop prediction. **(d)** Comparative SHAP analysis highlighting the relative importance of soil nutrient and climatic parameters in determining crop suitability.

The final figure of the SHAP decision plots shows how the nutrient and climatic factors affect the crop suitability predictions from the model proposed. The overall contribution of all the features to all the crop samples is shown in Figure 6a. The most important input variables are Phosphorus (P), humidity, Potassium (K), and rainfall. The contribution of Potassium (K = 47.0) and Phosphorus (*p* = 17.0) to the model output is positive while humidity (94.73%) and rainfall (26.31 cm) add strength to the prediction.

The prediction pathway of muskmelon is shown in Figure 6b. The dominant factors were potassium (K = 47.0) and Phosphorus (*p* = 17.0) with humidity (94.73%) and rainfall (26.31 cm) as the supporting factors. Based on these results, the model relates these nutrient and environmental conditions to muskmelon suitability.

Figure 6c shows the decision plot for apple prediction using SHAP. Potassium (K) and Phosphorus (P) are the second and third most important factors affecting the model output, both with a positive impact, while the highest positive impact is rainfall (26.31 cm). This indicates that moisture condition is a key factor involved in the model predicting watermelon production.

The prediction pathway for muskmelon is shown in Figure 6d. Along with Iron (Fe = 80.0), humidity, temperature (29.49 °C) and pH, micronutrients like Copper (Cu = 0.43) and Magnesium (Mg = 14.7) are important in predicting. The model thus identifies micronutrient availability and environmental conditions as important factors affecting apple suitability.

In general, the SHAP decision plots offer clear visualization of how the prediction process works, and they offer an explanation of which variables (nutrients and climatic) contribute to the crop recommendation. The results highlight the machine learning framework proposed to accurately capture the complex interactions of nutrient–climate and facilitate explainable crop prediction.

#### 5.3.3 SHAP dependency analysis

**Figure 7:**
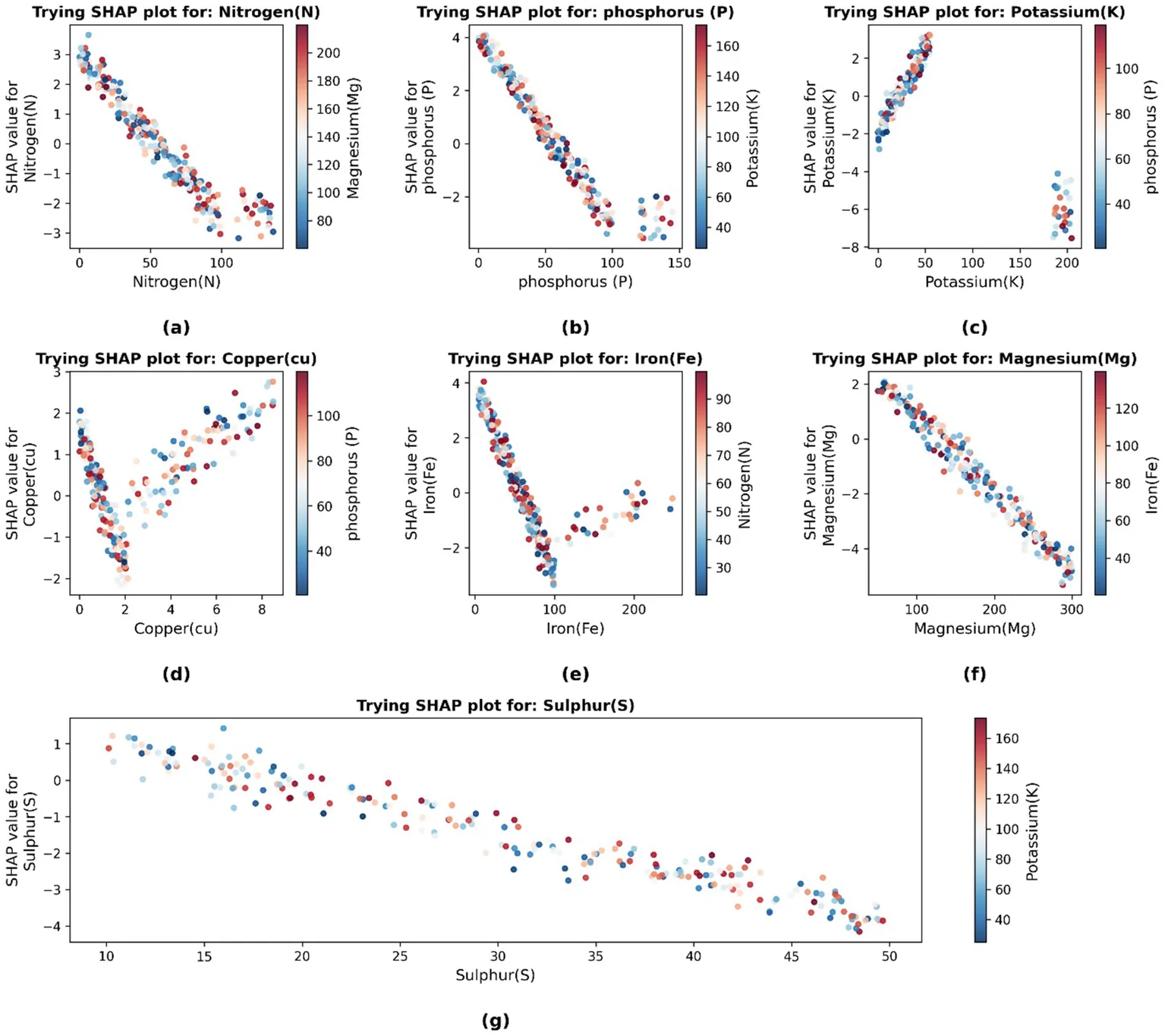
SHAP dependence plot analysis of soil nutrient features and their interactions in crop recommendation: **(a)** Nitrogen (N), **(b)** Phosphorus (P), **(c)** Potassium (K), **(d)** Copper (Cu), **(e)** Iron (Fe), **(f)** Magnesium (Mg), and **(g)** Sulphur (S), illustrating the relationship between nutrient values and their SHAP contributions, with colour gradients representing interactions with other influential nutrient features.

SHAP dependence plots show the relationship between feature values and their contribution to the model prediction, highlighting how each feature contributes to the overall prediction. The dependency plots for six of the most important features are shown in Figure 7, where the colour of the plot represents the second feature which interacts with the feature shown on the X axis.

In Figure 7a the correlation between Nitrogen (N) is clearly negative; higher amounts of Nitrogen (N) consequently result in increasingly lower values of model output. Magnesium (Mg) is a secondary interacting factor (high or low levels in the nitrogen range). Phosphorus (P) is decreasing in Figure 7b, with higher concentrations having lower SHAP values, which is a very strong negative relationship. Potassium (K) interacts with Phosphorus, and a separate secondary cluster can be seen at P levels of 120–150, which is related to high levels of Potassium. However, Potassium (K) showed a non-linear trend in Figure 7c with two major trends, one around low K (0–50) and the other around high K (200), indicating the effect of K changes may be large depending on the concentration and mostly driven by Phosphorus (P).

Copper (Cu) has a distinct threshold effect, with its SHAP value staying at around zero or negative at very low concentrations of Cu (when Cu < 1), but rising gradually as Cu increases further, with Phosphorus (P) driving this positive change at high concentrations (see Figure 7d). In Figure 7e, at higher concentrations, the predictions worsen with the SHAP values decreasing with increasing Fe. The secondary interacting factor is Nitrogen (N) and those of high Nitrogen (pink) values have greater negative impacts. In Figure 7f, Magnesium (Mg) has a consistent decreasing SHAP trend as it increases above 100, decreasing significantly at high concentrations. Iron (Fe) is the secondary interacting factor where low Fe values (blue) predominate in high Magnesium levels. In Figure 7g the concentration of Sulphur (S) is increasing from 10 to 50 and the trend is negative as the concentration is increasing the values are going down. The secondary interacting factor is Potassium (K) with low Potassium values (blue) indicating the highest negative interactions at higher levels of Sulphur.

The dependency plots are used in combination to reveal the interactions between nutrients and nutrient thresholds that influence the prediction of crops and will support the development of optimal nutrient recommendations and better crop suitability assessment.

#### 5.3.4 LIME interpretation

The goal of this section is to achieve clear understanding of the created model at the local level. This section aims to ensure local model interpretability. LIME identifies the main factors affecting individual crops and explains individual crop predictions. Phosphorus (P), Iron (Fe), Humidity, Magnesium (Mg), and Nitrogen (N) are significant contributors as shown in the visualization. Exceeding nutrient thresholds have a negative influence on suitability, confirming that thresholds have an effect on predictions (P and Fe). This will increase transparency of artificial intelligence recommendations, supporting farmers to optimise nutrient management to select crops.

**Figure 8:**
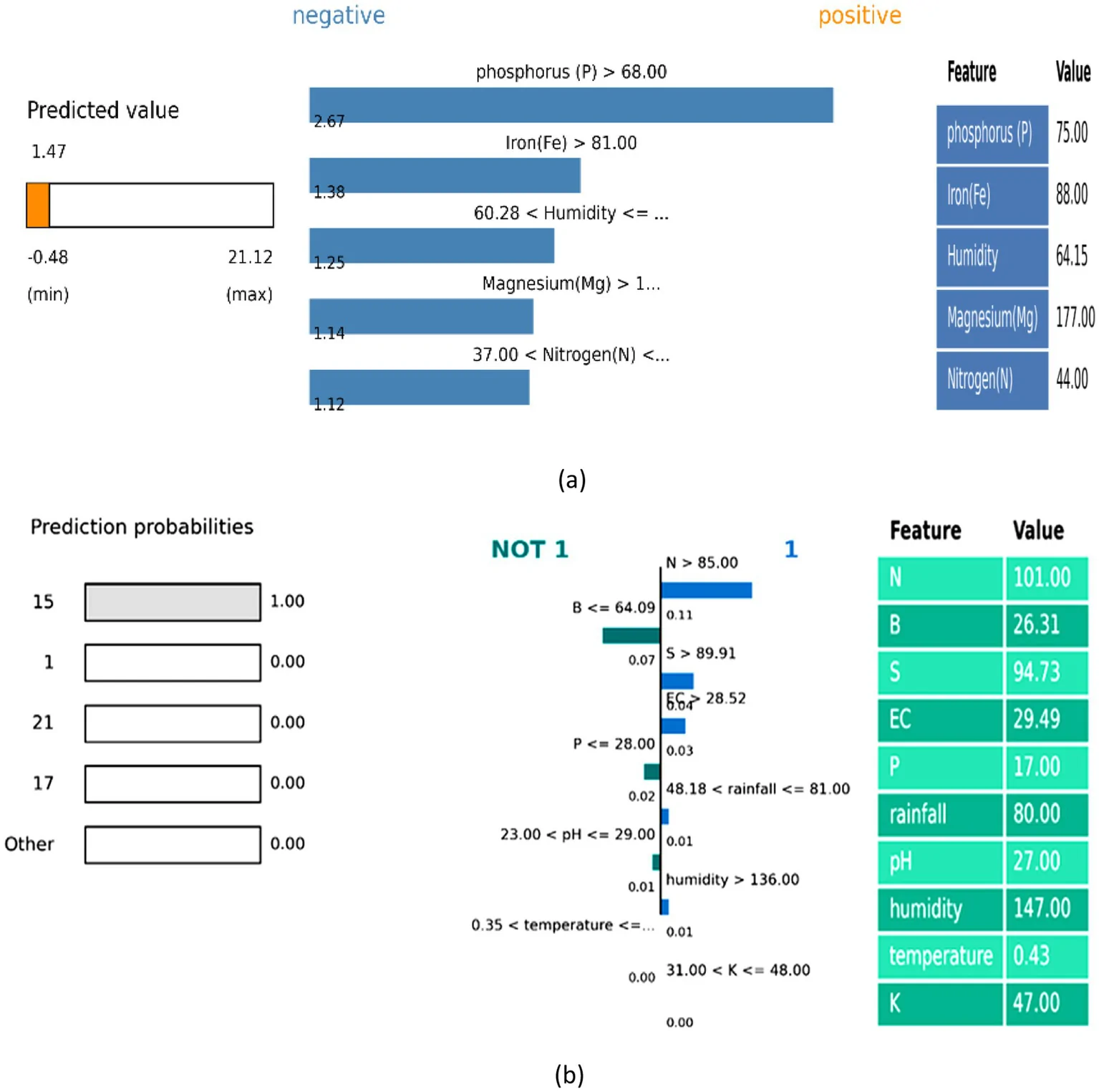
**(a)** LIME explanation for a sample prediction: feature contributions and corresponding values. **(b)** LIME explanation for class prediction showing prediction probabilities and feature contributions.

The LIME bar chart in Figure 8a reveals that all the features shown here have negative contribution (blue bars) to the crop prediction. The key negative conditions are: Phosphorus (P) > 68.00, Iron (Fe) > 81.00, Humidity > 60.28, Magnesium (Mg) > 1, and Nitrogen (N) within the range 37.00 < N. The actual feature values from the data instance are: *N* = 101.00, B = 26.31, S = 94.73, EC = 29.49, *p* = 17.00, rainfall = 80.00, pH = 27.00, humidity = 147.00, temperature = 0.43, and K = 47.00. The values prove that multiple features are above the negative threshold values, having significant impact on the prediction result.

For this model, as shown in the Figure 8b Class 15 is predicted with full confidence (probability = 1.00) and the other classes have zero probability. The decision rules highlight key features influencing this prediction: Nitrogen (N > 85.00), Boron (B ≤ 64.09), Sulphur (S > 89.91), rainfall (48.18 < rainfall ≤ 81.00), pH (23.00 < pH ≤ 29.00), humidity (> 136.00), and temperature (0.35 < temperature). The model classification is verified by the actual values *N* = 101.00, B = 26.31 and S = 94.73 as per the decision rules. LIME describes the feature importance of the recommendation, providing valuable information to the farmer to make adjustments to the conditions for optimal crop selection.

### 5.4 Proposed KHO-based explainable crop recommendation framework

The baseline models were then evaluated and the results of their prediction were interpreted with XAI techniques, which was then fed into the Tab Transformer model to come up with the suggested crop recommendation framework, Crop Recommendation Framework–KHO. KHO was used to optimize nutrient parameters and climatic parameters, SHAP and LIME were used to give transparent explanation of the optimized predictions. The integrated framework enhances the clarity of crop recommendations and aids in making informed decisions for agriculture.

The proposed framework follows a sequential workflow in which the Tab Transformer first performs crop prediction using integrated nutrient and climatic features. Krill Herd Optimization is subsequently employed to generate optimized nutrient–climate profiles, while SHAP and LIME provide global and local explanations of the optimized predictions. This integrated workflow enables both accurate crop recommendation and transparent interpretation of the model decisions.

#### 5.4.1 KHO optimization process

The Krill Herd (KH) algorithm is a nature Inspired metaheuristic optimization technique that is inspired from the behavior of Antarctic Krill to fine-tune the parameters of crop recommendation. The objective of this study is to use KH optimization to find the best combination of nutrient and climatic conditions for each crop so as to maximize the prediction accuracy and recommendation quality. The algorithm is designed to converge to an optimum nutrient-climate scheme for specific crops by using an iterative adjustment process of the values of the various features, including nitrogen, phosphorus, potassium, iron and rainfall. This method helps ensure more accurate AI-driven crop recommendations and aligns the gap between AI predictions and farming decisions.

##### 5.4.1.1 KHO process steps

###### 5.4.1.1.1 Data preparation

The dataset of the crop is loaded, features (X) and target crop (y) are extracted and Min Max Scaler is used to normalize inputs so that the contribution of each feature is equal.

###### 5.4.1.1.2 Krill initialization

Krill initialised by placing positions at random, but the positions are normalized as combinations of nutrients and climate optimized by fitness tracking will be found iteratively.

###### 5.4.1.1.3 Fitness evaluation

A Decision Tree classifier is trained on each krill’s position and accuracy is computed. The fitness value (1-Accuracy) is used to minimize; hence higher fitness values (higher accuracy) are favored.

###### 5.4.1.1.4 Krill movement

The position of each krill is determined by the current position, best known solution, and random drift, but then clipped to the [0,1] normalized range.

###### 5.4.1.1.5 Optimization and output

Once convergence is achieved the optimum values of the krill are normalized back to their original scale and presented in a bar chart as the optimized nutrient-climate profile for the chosen crop.

#### 5.4.2 Optimized nutrient and climate profiles

**Figure 9:**
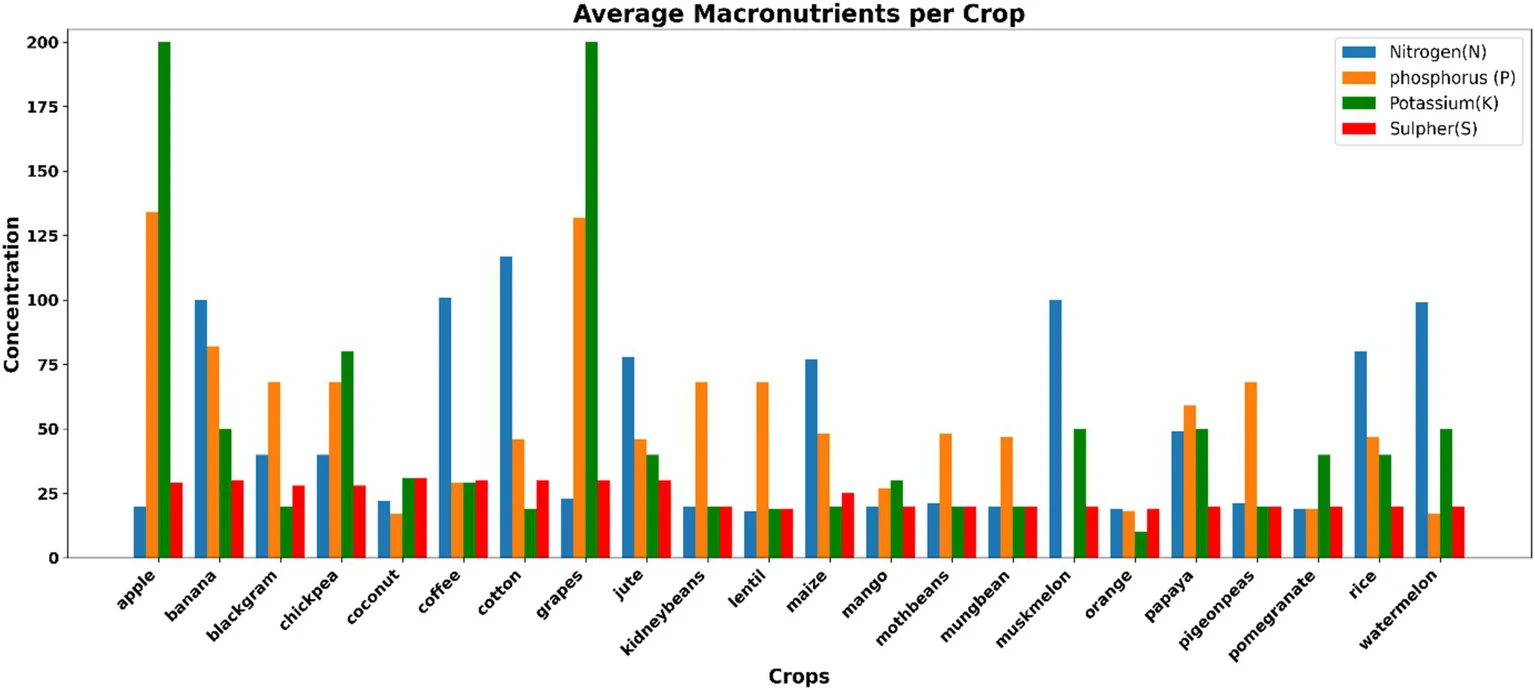
Comparative distribution of macronutrients (N, P, K, S) across different crop types.

Figure 9 visually compares key macronutrients — Nitrogen (N), Phosphorus (P), Potassium (K), and Sulphur (S) — across 22 crops including apple, banana, grapes, cotton, and maize. Grapes and cotton show the highest Potassium (K) concentrations (~200), while crops like muskmelon and rice show notably high Nitrogen (N) levels. This comparison helps farmers and researchers identify crop-specific nutrient needs, enabling precise fertilization that reduces costs and prevents soil degradation. Understanding these nutrient patterns also supports better crop rotation planning, maintaining soil fertility and reducing the risk of nutrient depletion. Overall, this visualization serves as a valuable decision-making aid for optimizing agricultural productivity while minimizing environmental impact. These optimized nutrient–climate profiles provide the basis for the explainability analysis presented in the subsequent section, thereby linking optimization with interpretable crop recommendation.

**Figure 10:**
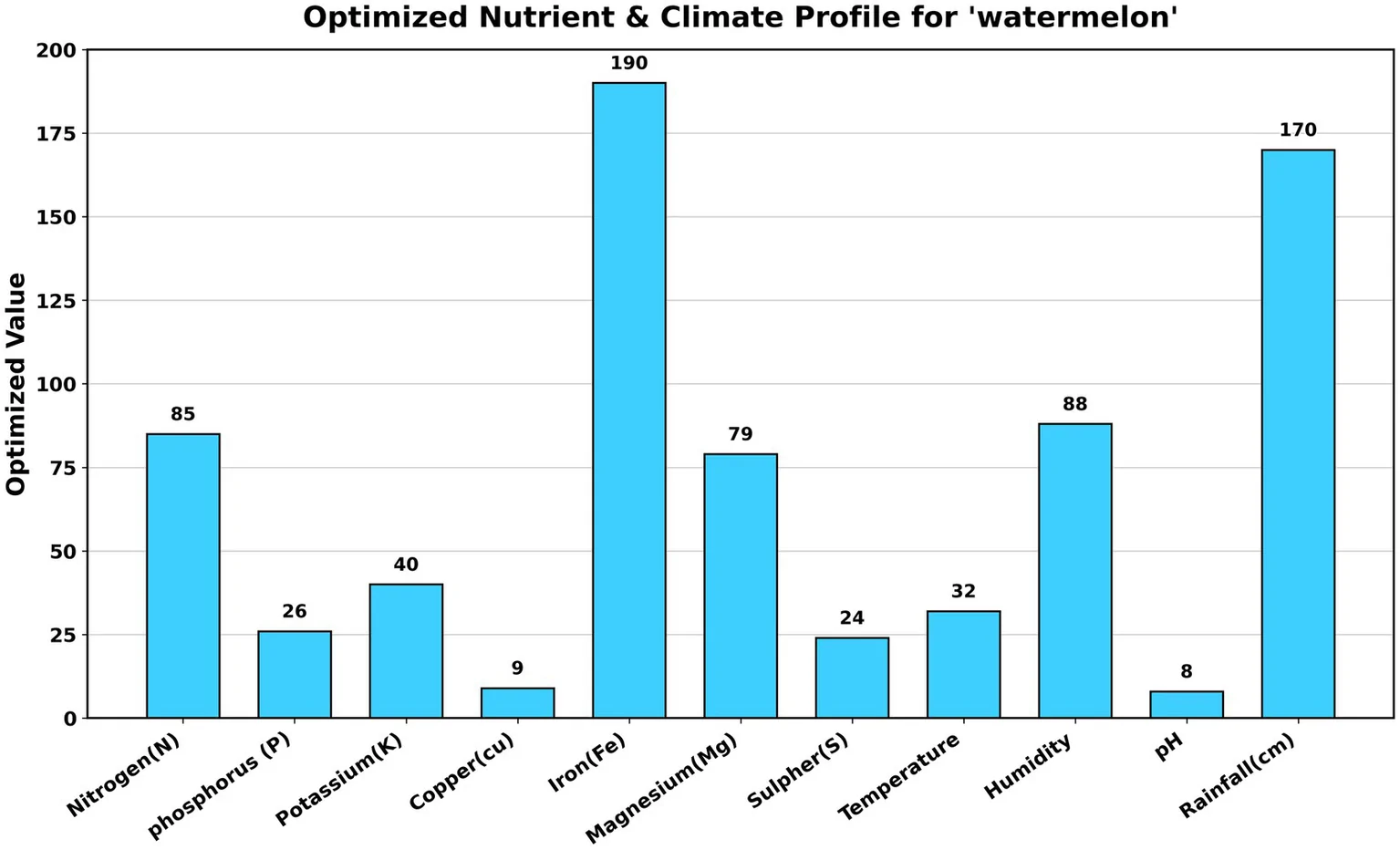
Model-recommended optimal nutrient and climatic conditions for watermelon cultivation.

Figure 10 illustrates the optimized nutrient and climate profile for watermelon cultivation derived using the Krill Herd metaheuristic approach. Iron (Fe) has the highest optimized value (~190), followed by Rainfall (~170) and Nitrogen (N) (~85). Magnesium (Mg) and Humidity also show relatively high optimized values. In contrast, Copper (Cu) and pH have the lowest optimized values (~10), suggesting they are required in smaller quantities or within a narrow range for optimal watermelon growth. Phosphorus (P), Potassium (K), Sulphur (S), and Temperature fall in the mid-range of optimized values. This profile provides insights into the ideal environmental and nutritional conditions for maximizing watermelon yield and quality.

**Figure 11:**
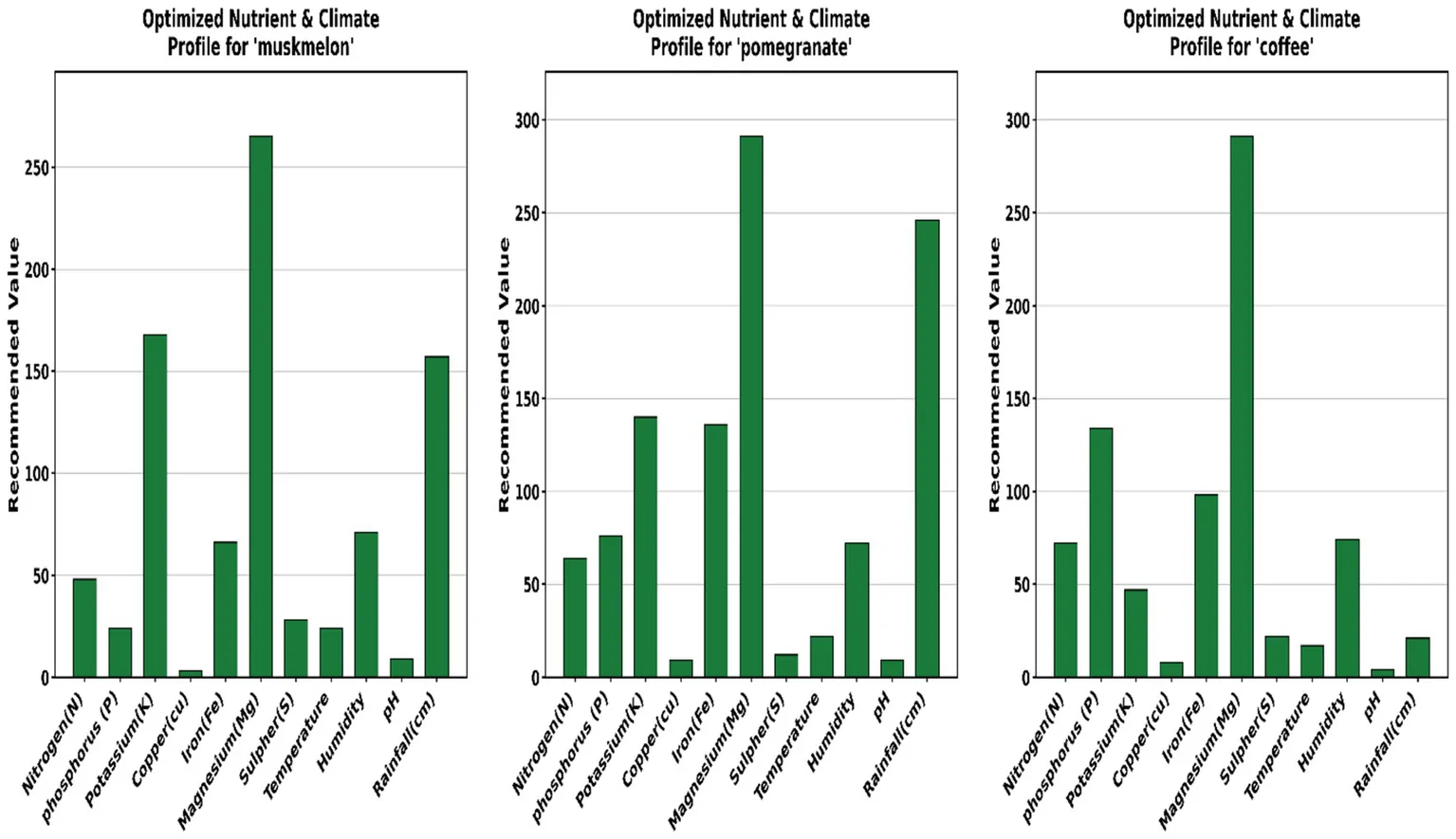
Optimized nutrient and climate profiles for Muskmelon, Pomegranate, and Coffee.

The nutrient/ climate profile optimization is done using the Krill Herd Optimization (KHO) algorithm to find the optimal nutrient and climate profile for each crop. When a crop is selected from the interactive dropdown it will display a bar chart of recommended values for Nitrogen (N), Phosphorus (P), Potassium (K), Copper (Cu), Iron (Fe), Magnesium (Mg), Sulphur (S), Temperature, Humidity, pH and Rainfall (cm) as seen in Figure 11. This visualization can assist precision agriculture by monitoring crop specific nutrient needs, assisting in resource allocation, maximizing yield, and providing farmers with the information they need when making crop selection decisions.

For Muskmelon, Magnesium (Mg) has the highest recommended value (~265) followed by Potassium (K) (~165) and Rainfall (~155) as shown in Figure 11. The values of Iron (Fe) (~68) and Humidity (Hum) (~75) are moderately high, whereas those of Copper (Cu) and pH are extremely low (~5).

For Pomegranate, in Figure 11, the magnesium (Mg) accounts for ~290, followed by Rainfall (~245) and Iron (Fe) (~138). Moderate contributions are given by Potassium (K) (~142) and Phosphorus (P) (~78); while Copper (Cu) and pH are the lowest (~10).

For Coffee, the highest recommended concentrations are for Mg (~290), P (~135) and Fe (~100) in Figure 11. Nitrogen (~73) and Humidity (~75) exhibit moderate value, whereas, Rainfall (~20), Temperature (~15) and pH (~5) need low concentration.

Across the three crops Magnesium (Mg) is the most critical nutrient, and pH and Copper (Cu) are consistently the lowest needs. The cross-crop comparison confirms the ability of the KHO algorithm to produce optimized nutrient and climate profiles for individual crops, enabling precision farming practices.

#### 5.4.3 KHO-based explainable prediction

**Figure 12:**
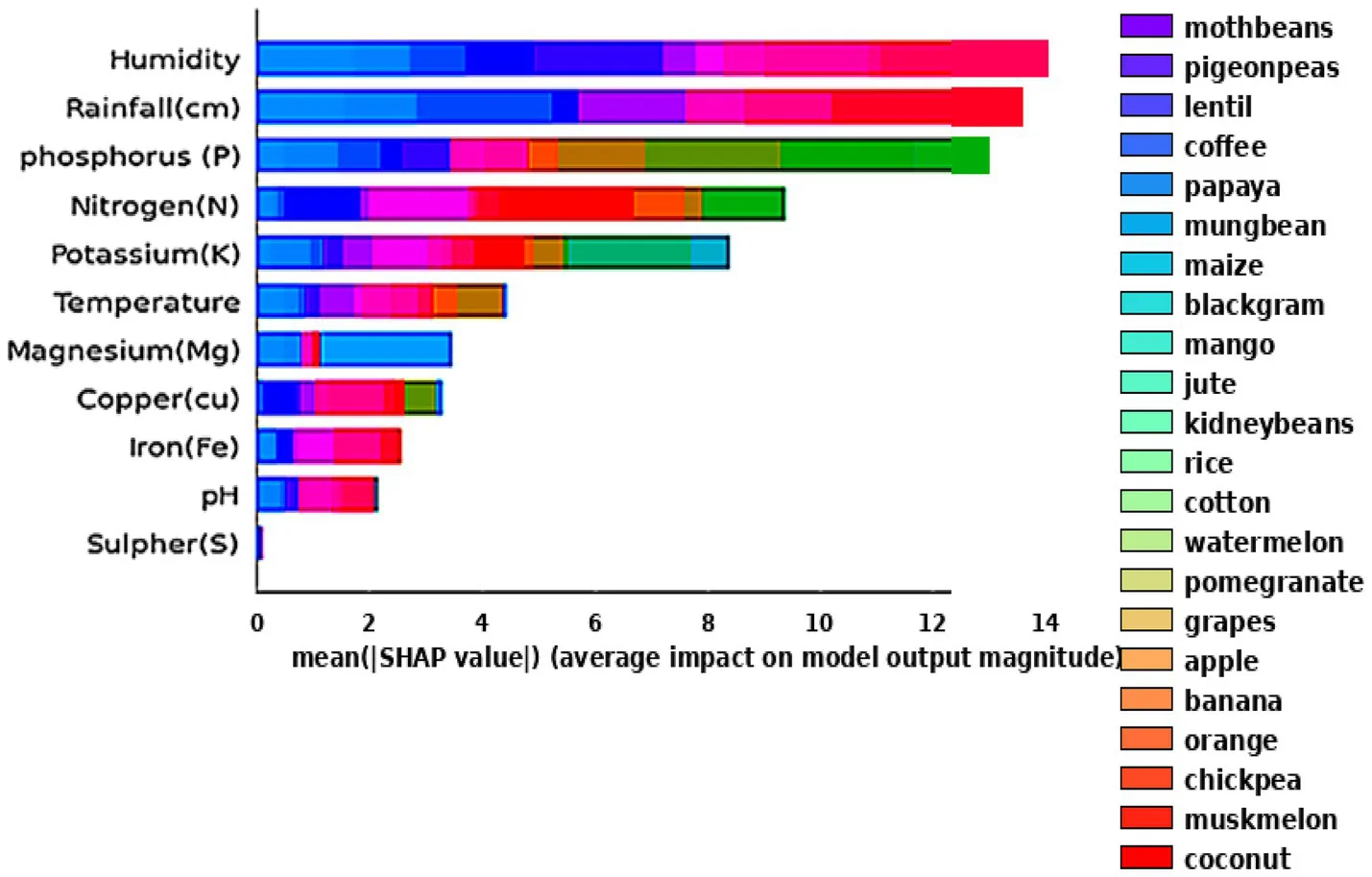
Global SHAP-based interpretation of feature contributions across 22 crop classes for crop suitability prediction.

Figure 12 displays the SHAP summary plot where the features’ impacts on the crop predictions are summarized for all 22 crops. The most prominent features are Humidity with the highest mean absolute SHAP value and Rainfall. In addition, phosphorus (P) and nitrogen (N) are important contributors, with Sulphur (S) and pH exhibiting the least impact. The colour coded bars show the relative contributions of the various crops such as mothbean, pigeonpea, lentil, coffee and papaya to the features, so that one can have a general overview of the feature importance across different types of crops.

**Figure 13:**
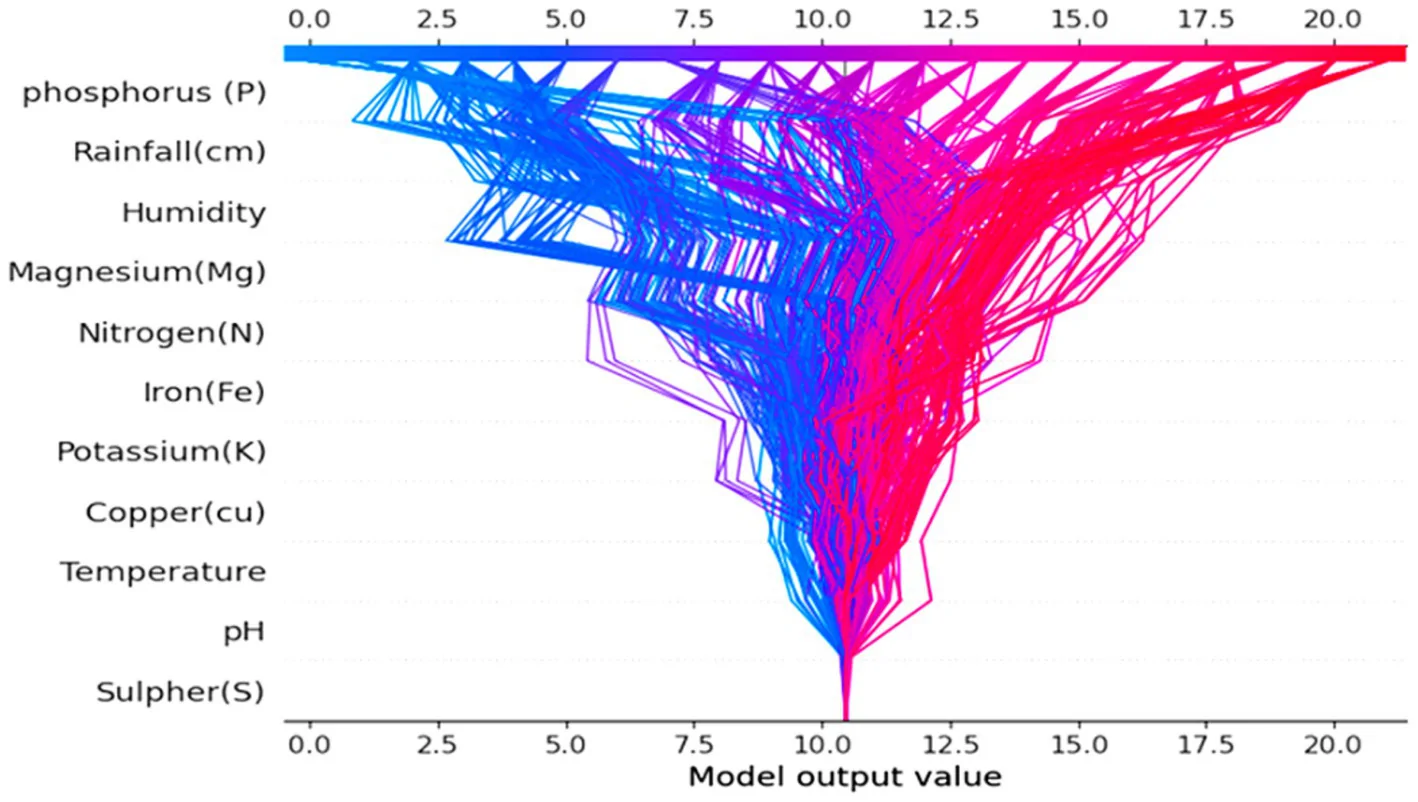
SHAP decision plot for multiple samples – Crop suitability analysis.

For KHO-SHAP, the Decision plot shown in Figure 13 shows that the most influential feature is Phosphorus (P) followed by Rainfall and Humidity. The colour gradient shows the “direction of the tendency” for the feature values to produce predictions towards particular crops, from blue (low model output) to red (high model output). High Phosphorus and Rainfall affect crop suitability in a positive way, whereas low Magnesium and low pH affect crop suitability in a negative way.

**Figure 14:**
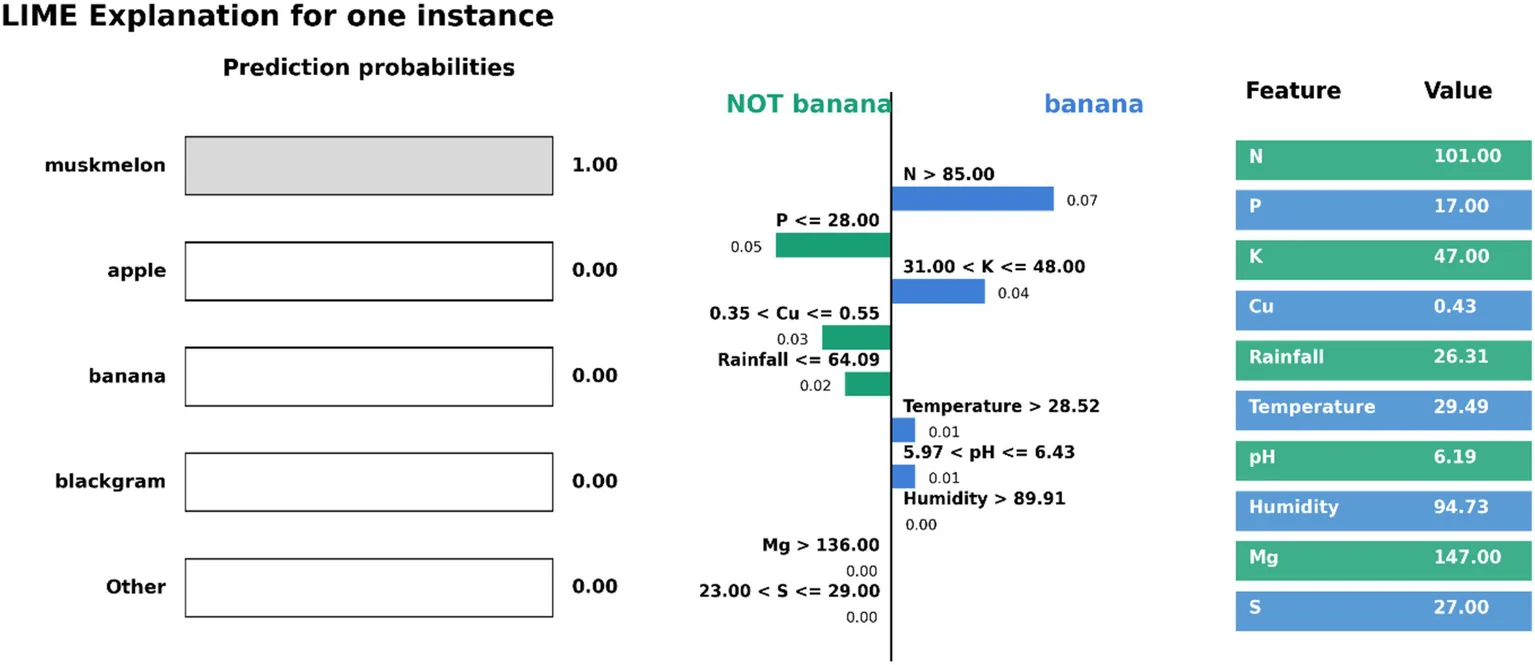
LIME-based local interpretability analysis for a single crop prediction.

Figure 14 illustrates the LIME explanation for a single case where the KHO-tuned model is predicting muskmelon with a 100% confidence. Key decision rules include *N* > 85.00, *p* ≤ 28.00, and 31.00 < K ≤ 48.00. The real values of the feature (*N* = 101, *p* = 17, K = 47, Cu = 0.43, and Rainfall = 26.31) meet the following criteria and classify the muskmelon. This transparency enables farmers to comprehend and rely on the model’s suggestions. Together, SHAP and LIME complement the KHO-optimized Tab Transformer model by providing global feature importance and local prediction explanations, respectively. This integrated explainability framework enables users to understand both the overall influence of nutrient and climatic factors and the reasoning behind individual crop recommendations, thereby improving the transparency, reliability, and practical applicability of the proposed crop recommendation system.

### 5.5 Evaluation of the proposed framework

This section evaluates the optimization performance of the proposed Krill Herd Optimization (KHO)-based crop recommendation framework by comparing its convergence behavior with other classical metaheuristic algorithms, namely Genetic Algorithm (GA), Particle Swarm Optimization (PSO), and Simulated Annealing (SA). The comparison demonstrates the effectiveness and convergence characteristics of KHO in the proposed framework.

#### 5.5.1 Comparison of KHO with classical optimization algorithms

**Figure 15:**
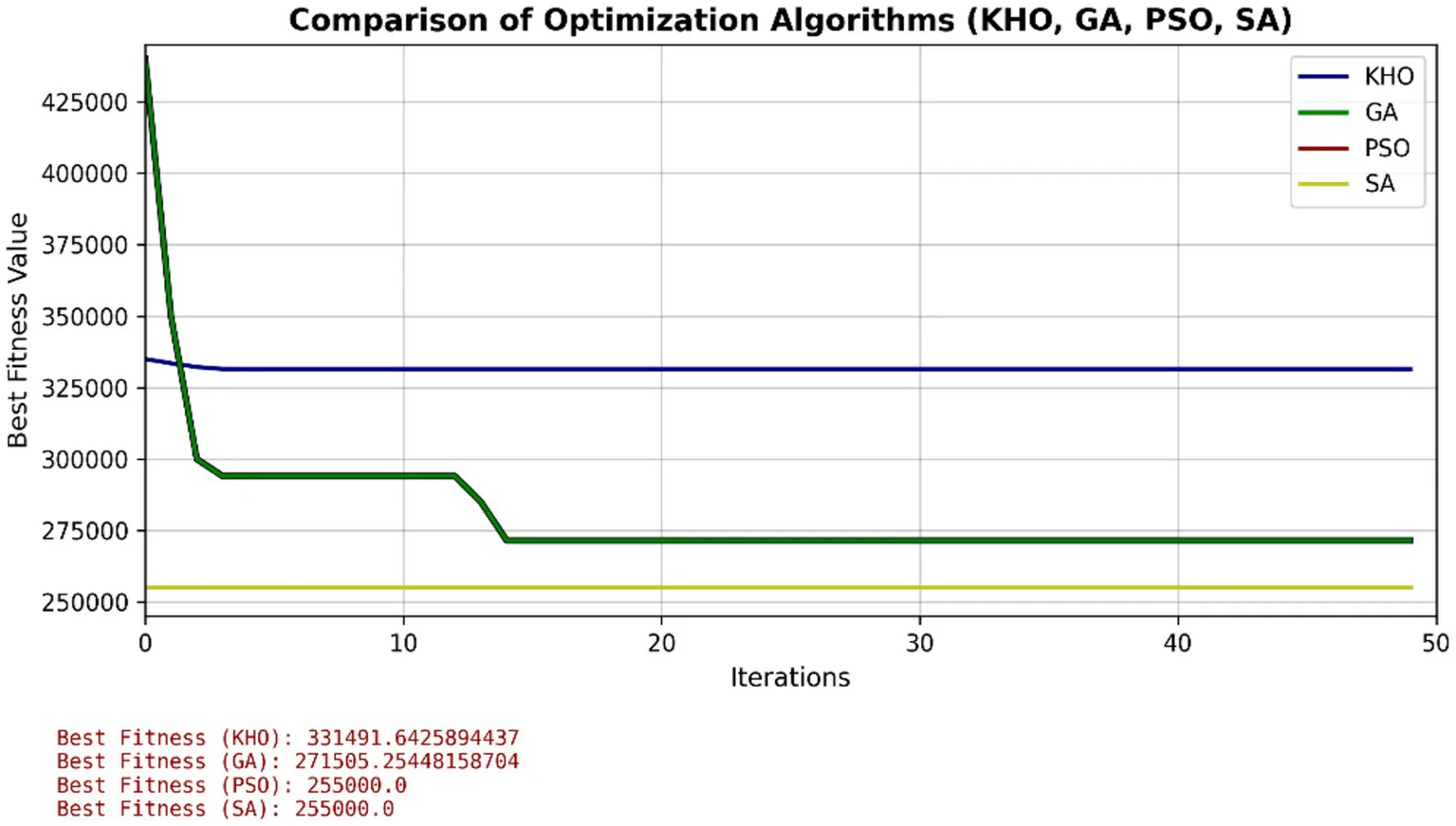
Convergence behavior and best fitness value comparison of Krill Herd Optimization (KHO), Genetic Algorithm (GA), Particle Swarm Optimization (PSO), and Simulated Annealing (SA) across iterations.

The convergence behavior of the optimization algorithms was checked by comparing the algorithms KHO, GA, PSO and SA for 50 optimization iterations and plotted in Figure 15. The smallest fitness value was obtained by PSO and SA, which was 255,000.0, thus exhibiting the best optimization results in terms of the fitness function chosen. The fitness value of GA converged to 271,505.25 and the fitness value of KHO converged to 331,491.64 and showed a stable convergence pattern during the optimization process. While the KHO did not yield the best fitness value, it was chosen to be incorporated into the proposed framework since it offered a strong hyperparameter optimization which enhanced the overall predictive performance of Tab Transformer model. In addition, KHO allowed the creation of representations that took into account the nutrients and climate, in addition to the explainability analysis carried out with SHAP and LIME. Thus, the choice of KHO was not made due to the optimization fitness value but because of its role in the overall effectiveness, robustness, and interpretability of the proposed crop recommendation framework.

## 6 Conclusion

This study presented an intelligent and explainable crop recommendation framework that integrates Tab Transformer, Krill Herd Optimization (KHO), and Explainable Artificial Intelligence (XAI) techniques to support accurate and transparent decision-making in precision agriculture. The proposed framework utilizes macronutrients, micronutrients, and environmental parameters to effectively capture complex relationships among agricultural features and generate reliable crop recommendations.

The experimental results demonstrated that the proposed Tab Transformer-based framework achieved superior crop classification performance compared with the baseline models. In addition, regression analysis was conducted to further evaluate the predictive capability of the learning models using continuous evaluation metrics. In addition, regression analysis was conducted to further evaluate the predictive capability of the learning models using continuous evaluation metrics. The model achieved the highest classification accuracy of 99% and superior regression performance with an MAE of 0.10, MSE of 0.01, and an R^2^ score of 0.98. Furthermore, integrating nutrient and climatic features improved crop discrimination compared with using macronutrients alone. The explainability analysis using SHAP and LIME consistently identified humidity, phosphorus, potassium, rainfall, and nitrogen as the most influential factors affecting crop recommendations, thereby improving the transparency and reliability of the prediction process.

The proposed framework combines transformer-based learning, optimization, and explainable AI within a unified architecture. The self-attention mechanism of the Tab Transformer effectively learns complex feature interactions, while KHO generates optimized nutrient and climate profiles for crop recommendation. In addition, SHAP and LIME provide interpretable explanations of model predictions, enabling users to understand the contribution of individual nutrient and environmental factors. These capabilities make the proposed framework a valuable decision-support tool for farmers, agricultural experts, and policymakers by improving crop selection, optimizing resource utilization, reducing unnecessary fertilizer application, and promoting sustainable agricultural practices.

The present study has a few limitations, however, with promising performance. The suggested framework was tested with only one publicly available benchmark data set from Mendeley repository that might be not representative across a wide geographical area, soil properties and climatic conditions. Moreover, the framework was designed and tested with the static benchmark data, and it cannot be implemented in real time in the smart agriculture environment. Future work will be focused on developing real time data acquisition mechanisms which include the sensor network soil sensors, automated weather stations and dynamic climate forecasting services, to improve the soil and environment state information for providing adaptive crop recommendation for practical use in smart agriculture. While the benchmark dataset selected for this study serves as a good foundation to build models and make comparisons, more validation of the robustness, scalability, generalization ability and practical applicability of the proposed framework with large-scale real-world datasets from various agricultural settings is needed to further assess the framework’s performance.

## 7 Future work

To further improve the accuracy of crop recommendation, the proposed framework will be extended in future by adding more agricultural, environmental, and geographical parameters such as soil moisture, solar radiation, wind speed, geographic information, etc., which will improve the representation of the features. The adoption of real-time IoT-based sensor networks and dynamic weather prediction models will be discussed – to continuously monitor soil and weather conditions and provide crop suggestions based on location and time in a smart agriculture application. Additionally, there is potential for employing more sophisticated optimization methods and hybrid optimization strategies to enhance computational efficiency and model adaptability. Finally, the proposed framework will be tested with large-scale and geographically distributed agricultural data sets and implemented in actual precision farming settings, proving the scalability, robustness, and applicability of the framework.

## Data Availability

Publicly available datasets were analyzed in this study. This data can be found here: https://www.kaggle.com/datasets/manikantasanjayv/crop-recommender-dataset-with-soil-nutrients.
